# Polaritonic linewidth asymmetry in the strong and ultrastrong coupling regime

**DOI:** 10.1515/nanoph-2023-0492

**Published:** 2023-10-18

**Authors:** Adriana Canales, Therese Karmstrand, Denis G. Baranov, Tomasz J. Antosiewicz, Timur O. Shegai

**Affiliations:** Department of Physics, Chalmers University of Technology, 412 96, Göteborg, Sweden; Department of Microtechnology and Nanoscience (MC2), Chalmers University of Technology, 412 96, Göteborg, Sweden; Center for Photonics and 2D Materials, Moscow Institute of Physics and Technology, Dolgoprudny 141700, Russia; Faculty of Physics, University of Warsaw, Pasteura 5, 02-093 Warsaw, Poland

**Keywords:** strong coupling, ultrastrong coupling, polaritons, Fabry–Pérot microcavity, plasmonic resonance, meta-atoms

## Abstract

The intriguing properties of polaritons resulting from strong and ultrastrong light–matter coupling have been extensively investigated. However, most research has focused on spectroscopic characteristics of polaritons, such as their eigenfrequencies and Rabi splitting. Here, we study the decay rates of a plasmon–microcavity system in the strong and ultrastrong coupling regimes experimentally and numerically. We use a classical scattering matrix approach, approximating our plasmonic system with an effective Lorentz model, to obtain the decay rates through the imaginary part of the complex quasinormal mode eigenfrequencies. Our classical model automatically includes all the interaction terms necessary to account for ultrastrong coupling without dealing with the rotating-wave approximation and the diamagnetic term. We find an asymmetry in polaritonic decay rates, which deviate from the expected average of the uncoupled system’s decay rates at zero detuning. Although this phenomenon has been previously observed in exciton–polaritons and attributed to their disorder, we observe it even in our homogeneous system. As the coupling strength of the plasmon–microcavity system increases, the asymmetry also increases and can become so significant that the lower (upper) polariton decay rate reduction (increase) goes beyond the uncoupled decay rates, *γ*_−_ < *γ*_0,*c*_ < *γ*_+_. Furthermore, our findings demonstrate that polaritonic linewidth asymmetry is a generic phenomenon that persists even in the case of bulk polaritons.

## 1 Introduction

The behavior of confined electromagnetic modes and resonant material excitations, such as excitons and phonons, is heavily influenced by the strength of their interaction. While previous research in the weak coupling regime has focused on modifying the emitter’s decay rate [1–3], there has been less emphasis on the decay rates in the strong and ultrastrong coupling (USC) regimes. In situations where two hybrid light–matter states (polaritons) are formed in the strong coupling (SC) regime, the decay rates of both upper and lower polaritons are expected to equal the average of the uncoupled decay rates [4], with an asymmetry in decay rates only occurring at nonzero detuning [5]. However, experimental observations in exciton–polaritons in quantum wells revealed a notable reduction in the lower polariton linewidth [6–8]. The polaritonic linewidth asymmetry was explained through various effects related to the excitonic inhomogeneous broadening given by the disorder [9–11], including motional narrowing [6] and excitonic absorption asymmetry [7, 8]. More recently, works in other disordered materials beyond quantum wells have also reported a polariton linewidth narrowing, such as organic dyes [12, 13] and transition metal dichalcogenides [14].

Due to a limited amount of platforms that can span a broad range of light–matter interaction regimes, from weak to ultrastrong [15–17], there has yet to be a systematic study on the decay rates of polaritons covering various mode detunings and coupling strengths. Subwavelength optically resonant metallic nanoparticles, referred to as *meta-atoms* [18], have great potential to span all coupling regimes due to their large and tunable oscillator strength [19, 20]. Arranging such meta-atoms in lattices and coupling them to a cavity mode results in polaritonic modes, similar to the modes obtained using quantum emitters [21–26], but with a high degree of control and tunability. Thus, the meta-atoms enable a *model* for polaritons with the capacity to reach deep strong coupling [27], as well as to engineer chiral polaritonic states thanks to the availability of chiral meta-atom geometries [28–30]. Interestingly, this platform has shown a significant narrowing of the lower polariton (LP) and a broadening of the upper polariton (UP) in the USC regime [31], which inspired this study.

Here, we investigate the decay rates of polaritons in plasmon–microcavity systems as they are manipulated from the weak to the strong and ultrastrong coupling regimes. Our approach utilizes classical electromagnetic calculations with a pole-search technique to calculate the system’s eigenfrequencies. To simplify the pole-search method, we approximate the optical response of the gold nanodisk (meta-atom) arrays with an equivalent homogeneous thin film described by an effective Lorentz permittivity [19]. This removes the inhomogeneous broadening that was thought responsible for the linewidth narrowing in exciton–polaritons. Interestingly, we still find a polaritonic linewidth asymmetry with the LP (UP) decay rate remaining lower (larger) than the average of the uncoupled ones despite not having any disorder in the model. Even more so, we find that the LP decay rate may decrease beyond the uncoupled components. Although we focus on localized plasmons, this theoretical approach can be applied to any material whose dielectric response can be approximated with a Lorentzian permittivity. Moreover, we demonstrate that polaritonic linewidth asymmetry persists even for bulk polaritons. Our conclusions are, therefore, a consequence of this Lorentzian approximation and classical electromagnetism. This method enables exploring the decay rates of electromagnetic eigenstates in all coupling regimes, including weak, strong, and ultrastrong, without relying on simplified phenomenological models such as the commonly used Jaynes–Cummings model or effective coupled oscillator models using non-Hermitian Hamiltonians.

## 2 Results and discussion

### 2.1 Analysis of coupled plasmon–microcavity systems

Coupling Fabry–Pérot (FP) microcavities to meta-atoms offers the versatility to control the coupling strength by varying the geometrical parameters of the meta-atoms. The coupling strength can be enhanced by increasing the size of the meta-atoms or by increasing the density of the meta-atoms through changes in the pitch of the array, *ρ* ∝ Λ^−1^ [24]. Such versatility allows tuning the coupling from weak to ultrastrong in the same material platform. Here and throughout the manuscript, we specifically consider plasmonic nanodisks with variable nanodisk diameters, *d*, and heights, *h*.

**Figure 1:**
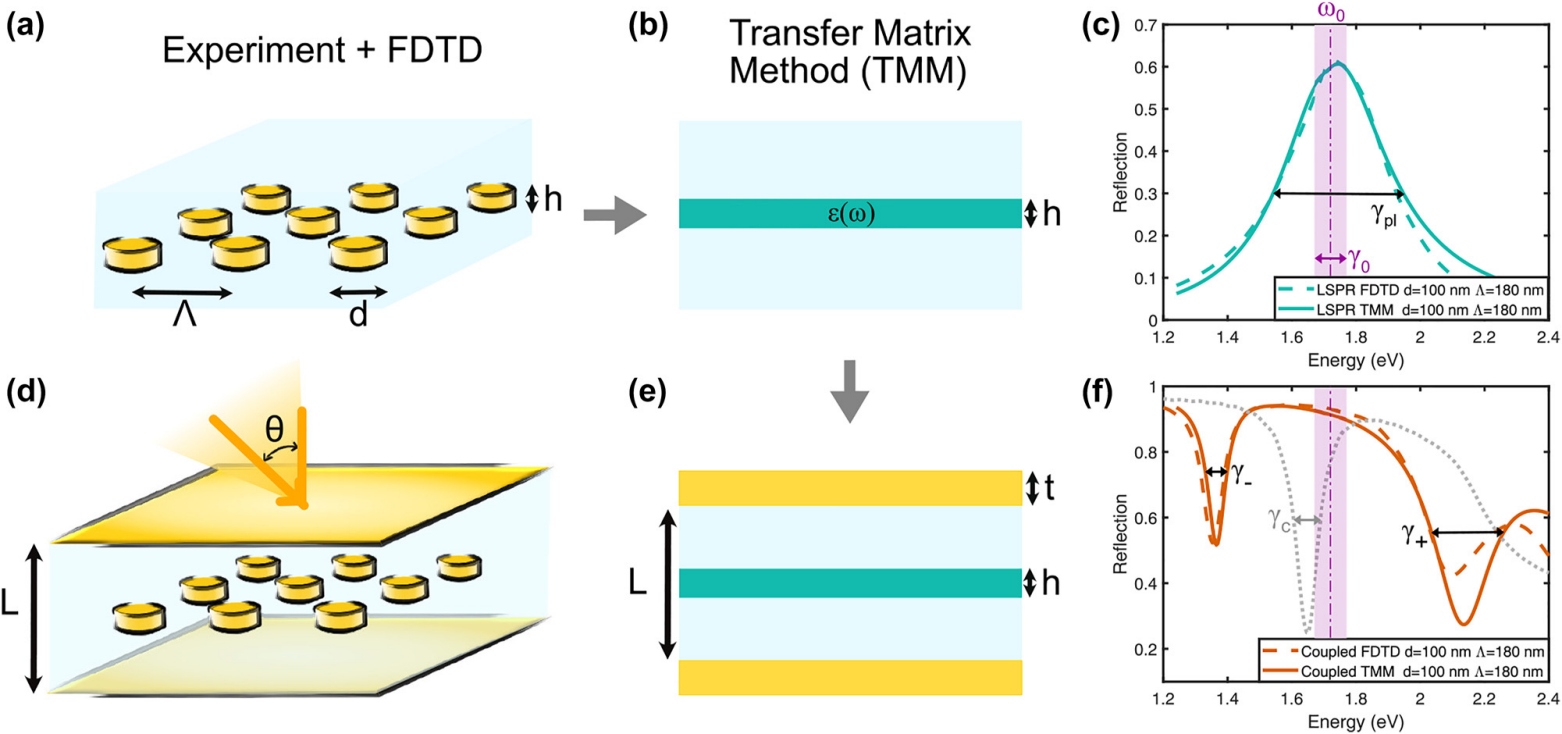
Schematic of the equivalent thin film analysis. Gold nanodisk (meta-atom) arrays in (a) glass and in (d) a gold microcavity. Each disk has a diameter, *d*, and height, *h*. The density of the array is given by its pitch (center-to-center), Λ. (b) To calculate the eigenfrequencies of the system, the nanodisk array was replaced with an equivalent thin film with an effective dispersive permittivity *ɛ*(*ω*), which has the same height as the disks and is surrounded by the glass. Then, it is embedded between two gold mirrors as in (e) to hybridize the plasmons with light. (c) Example of the reflection spectrum of a meta-atom in glass (*d* = 100 nm, Λ = 180 nm, and *h* = 20 nm) simulated by FDTD (dashed lines) and the slab with *ɛ*(*ω*) fitted via TMM (solid lines). The dash-dotted line shows the resonance frequency of the Lorentzian associated with the LSPR. The shaded area in purple corresponds to the *bare* plasmon decay rate, *γ*_0_, while the total decay rate of the plasmon in free space is *γ*_pl_. (f) Reflection of the same array coupled to a gold microcavity (*t* = 30 nm, *L* = 180 nm). The empty cavity reflection is shown in a gray dotted line. The decay rates of the polaritons are such that *γ*_−_ < *γ*_0,*c*_ < *γ*_+_.

The plasmonic nanodisk arrays in Figure 1a were fabricated by electron beam lithography (EBL), while the complete plasmon–microcavity coupled samples in Figure 1d were fabricated by additionally evaporating microcavity mirrors. The optical properties were measured by near-normal incidence reflection and angular dispersion in reflection mode by Fourier plane microscopy and spectroscopy (see Methods for further details).

The reflection spectra of the plasmonic nanodisk arrays at various incidence angles were simulated numerically with the finite-difference time-domain (FDTD) method using Ansys Lumerical software [32] as described in Methods. These simulations were used to guide the fabrication process and benchmark the theoretical analysis.

The decay rates and eigenfrequencies of the system were obtained simultaneously by a theoretical analysis based on a pole-search method [33] that uses the transfer matrix method (TMM) as described in Methods. This analysis requires an *analytical* description of the material’s permittivity. Therefore, we model plasmonic nanoparticle arrays as an equivalent thin homogeneous film characterized by a Lorentz permittivity, accounting for the localized surface plasmon resonance (LSPR) [34]:

(1) $$\varepsilon \left(\omega \right)=\varepsilon_{\infty }+\frac{f\omega_{P}^{2}}{\omega_{0}^{2}-\omega^{2}-\text{i}\gamma_{0}\omega },$$

where *ω*_0_ is the frequency of the plasmon resonance, $f\omega_{P}^{2}$ describes the coupling strength to the electromagnetic field through the oscillator strength of each nanodisk *f* and the density of the nanodisks contained in *ω*_*P*_. We considered *ɛ*_∞_ = 1.46^2^ as the background permittivity of the nanodisks’ surrounding medium (glass). Finally, *γ*_0_ describes all possible nonradiative decay channels in the resonant medium, such as ohmic loss, Joule loss, and dephasing. However, as will be discussed below, it does not include the radiative decay rate resulting from coupling to the electromagnetic field, governed by the parameter $f\omega_{P}^{2}$ [35].

The effective permittivity *ɛ*(*ω*) of each plasmonic array was determined by fitting the FDTD simulated reflection spectra of the bare array embedded in glass with the spectra obtained for an equivalent glass/Lorentz(*h*)/glass structure using the standard TMM as depicted in Figure 1b. The resulting parameters of effective permittivities for all studied plasmonic arrays are presented in Table S1 and Figure S1. Figure 1c shows one example of the simulated reflection spectrum for an array with *d* = 100 nm and Λ = 180 nm and the best fit of this spectrum by the effective Lorentz thin film model. Note that the homogeneous film approximation is only valid in the regime when the LSPR is not affected by the lattice modes [36, 37]. FDTD simulations confirmed that this is the case for the plasmonic arrays in this study. However, near-field interactions between particles are subsumed into the material properties.

The effective Lorentz permittivities were then used to calculate the reflection spectra of the coupled systems. This was done by adding more layers into TMM as shown in Figure 1e, – two layers for the gold mirrors with thickness *t* and two for the glass spacers (*n* = 1.46), each *L*/2 − *h*/2 thick. The permittivity of mirrors comprising the cavity was analytically described by a Drude–Lorentz model approximating the Johnson & Christy experimental data [38] (see Methods), to account for free-electrons response and interband transitions (IBTs) in gold.

A comparison between the results obtained by TMM and FDTD calculations, as depicted in Figure 1f, validates the efficiency of our method. Noteworthy, this approach remains effective even when dealing with high incident angles, as demonstrated by the comparison between Figure 4 and Figure S8. Discrepancies above 2 eV are likely due to modified interaction between explicit disks in a cavity and a homogenized film and the approximation of using a single Lorentzian pole to describe an inherently asymmetric LSPR of an array of disks. The equivalent thin film approximation holds for *s*-polarized light, but *p*-polarization requires fitting the effective permittivity for different angles to use a full anisotropic effective polarizability.

It is important to mention that Berkhout et al. [25] proposed a transfer matrix method to describe meta-atom arrays to investigate coupling with microcavities without resorting to the equivalent thin-film approximation. Unfortunately, our study experimentally examines angular dispersion, and we cannot use their model since it was derived for normal incidence. Our simplified Lorentzian approximation is compared to the method of Berkhout et al. [25] and discussed in Figure S2. Furthermore, our findings, in principle, are not limited to plasmonic arrays as described here but could have implications for other material platforms that exhibit strong light–matter coupling, including organic molecules, quantum dots, and transition metal dichalcogenides (TMDs), as long as a model Lorentzian permittivity accurately describes their optical responses.

### 2.2 Eigenfrequencies of the equivalent thin film

**Figure 2:**
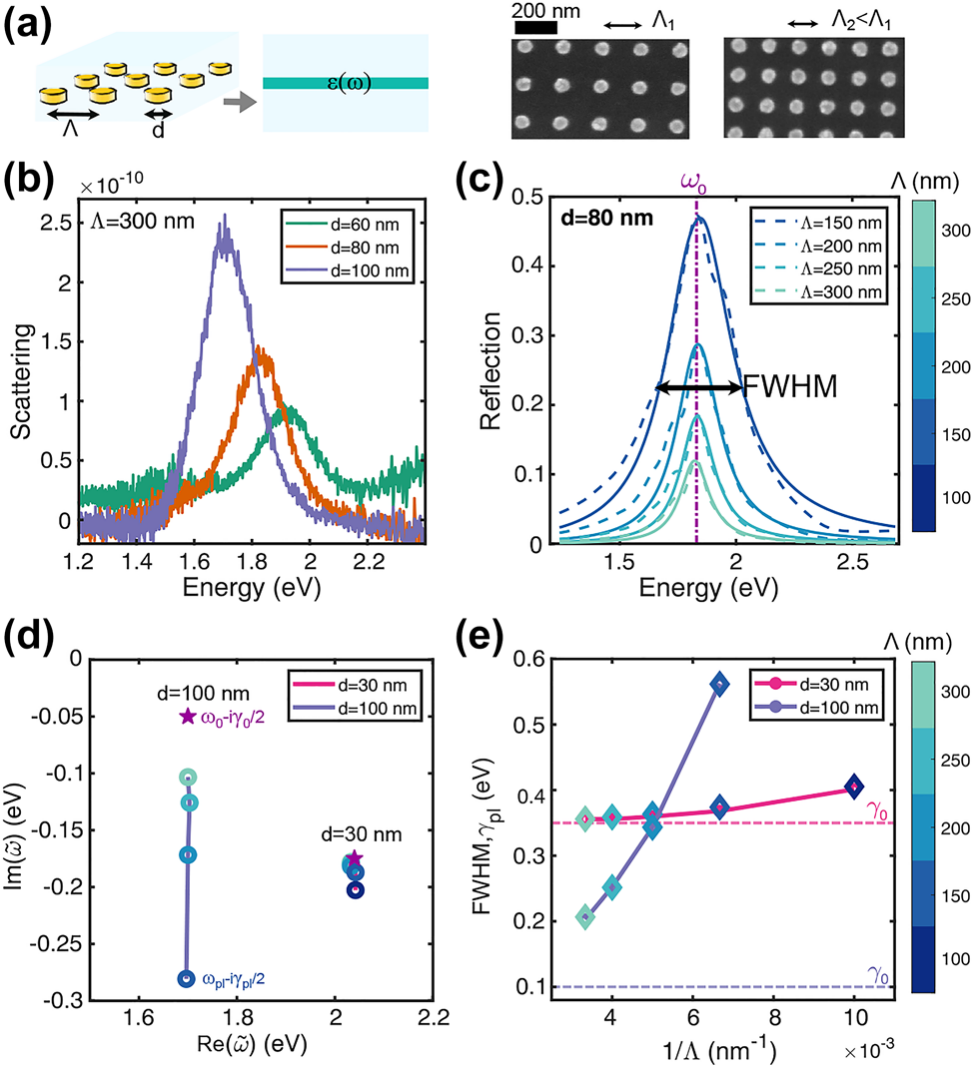
Plasmonic nanodisks outside the cavity. (a) Nanodisk array replaced with an equivalent thin film described by a Lorentzian permittivity and SEM images of two nanodisk densities (*d* = 60 nm, Λ_1_ = 180 nm, and Λ_2_ = 140 nm). (b) LSPR tuning observed by measured dark-field spectra of nanodisks with *d* = 60, 80, and 100 nm for a fixed pitch Λ = 300 nm. (c–e) Spectral dependence on the pitch Λ. (c) Comparison between FDTD simulated reflection spectra (dashed lines) of arrays with *d* = 80 nm for various pitches (colorbar) and reflection spectra of the corresponding equivalent Lorentzian thin films calculated using the TMM (solids lines). (d) Eigenfrequencies of the equivalent film in the complex-frequency plane for two diameters (*d* = 30 and 100 nm) and various pitches (colorbar). The stars mark the *bare* plasmon (effective Lorentzian thin film), *ω*_0_ − i*γ*_0_/2. (e) Resonant FWHM of the equivalent films obtained by fitting the reflection spectra in (c) coincides with the decay rates of the thin film QNMs, *γ*_pl_ (diamond markers with the pitch in the colorbar). The corresponding nonradiative decay rates *γ*_0_ of the effective media for both disk diameters are marked in dashed lines.

Prior to analyzing the coupled system, it is instructive to examine the equivalent Lorentzian film outside the cavity as in Figure 2a. Nanodisk arrays of various diameters and pitches were fabricated to tune the LSPR. The resonant energy of the LSPR is inversely proportional to the size of the nanodisk, and the dipole moment increases with the size of the nanodisk. As a result, larger particles exhibit greater scattering peaks at lower energies, as observed by dark-field spectroscopy in Figure 2b.

Increasing the array density, *ρ* ∝ Λ^−2^, enhances the coupling between plasmonic nanoparticles and the electromagnetic environment. Thus, despite having the same diameter, the arrays in the scanning electron microscope (SEM) images in Figure 2a have different effective coupling strengths. This increased coupling with the environment translates into higher reflection for smaller pitches, as shown in Figure 2c. When fitting FDTD with TMM, the dependence on Λ is contained in $f\omega_{P}^{2}$ while the resonant energy (*ℏω*_0_) and the bare decay rate (*γ*_0_) are constant within the studied parameter range as shown in Table S1.

The resonant frequencies of the bare equivalent thin films and the hybrid systems were found using the pole-search method [39, 40]. In this approach, the eigenfrequencies $\tilde{\omega }$ of an open optical system are found as poles of the scattering matrix eigenvalues on the complex-frequency plane. These eigenfrequencies form a complex-valued spectrum, $\tilde{\omega }=\omega -\text{i}\gamma /2$ , where *ω* represents a particular resonant frequency of a quasinormal mode (QNM), and *γ* represents its decay rate (or linewidth) [41]. Figure 2d shows the trajectories of the complex eigenfrequencies, denoted as *ω*_pl_ − i*γ*_pl_/2, of isolated equivalent films representing the nanodisk arrays with different pitches. Here, the plasmon’s decay rate is $\gamma_{\text{pl}}=2|Im\left(\tilde{\omega }\right)|$ .

The total decay rate of any optical resonator consists of the intrinsic (nonradiative) and the extrinsic (radiative) components. The total decay rate of the QNMs coincides with the full-width at half maximum (FWHM) of the reflectivity spectrum, as shown in Figure 2e. The nonradiative decay rate of the QNMs, *γ*_non-rad_, of the equivalent film is approximately given by the intrinsic decay rate of the effective medium, *γ*_non-rad_ ≈ *γ*_0_. The star symbols in Figure 2d represent the isolated (*bare*) plasmon frequencies, *ω*_0_ − i*γ*_0_/2, corresponding to a hypothetical situation when the nanodisks’ collective oscillations are fully shielded from the electromagnetic environment. Interestingly, smaller nanodisks have a higher nonradiative decay rate (corresponding to higher *γ*_0_ marked with dashed lines in Figure 2e) because their LSPR is spectrally closer to the onset of the IBTs of gold around 2.2 eV, as was previously observed for gold nanorods [42, 43].

The eigenfrequencies in the complex-frequency plane move downward from the bare plasmon, as observed in Figure 2d, due to the increase in the radiative decay rate because of the nanodisks’ density increase. Moreover, the nanodisks’ radiative coupling to the environment increases with its size [44]. Thus, as shown in Figure S3c, the coupling to the environment occurs through the term $f\omega_{P}^{2}$ [45], which considers the radiative decay increase due to the size (through *f*) and the density (through $\omega_{P}\propto \sqrt{\rho }$ ) of the nanodisks. Thus, smaller nanodisks tend to have frequencies closer to *ω*_0_ − i*γ*_0_/2 as demonstrated in Figure 2d for the two extreme diameters. Note, that within the studied parameter range the eigenfrequencies do not move significantly in the horizontal direction, so that *ω*_0_ remains nearly independent of Λ.

In what follows, we will consider nanodisks inside a resonant optical cavity. Hence, it is important to note that once the nanodisks are placed in the cavity, they interact with free space indirectly via the cavity. Therefore, as we will show below, the inherent *uncoupled* decay rate of the nanodisks, *γ*_0_, becomes the relevant characteristic of the nanoparticle array decay rate instead of the total *γ*_pl_.

### 2.3 Eigenfrequencies from weak to ultrastrong coupling

**Figure 3:**
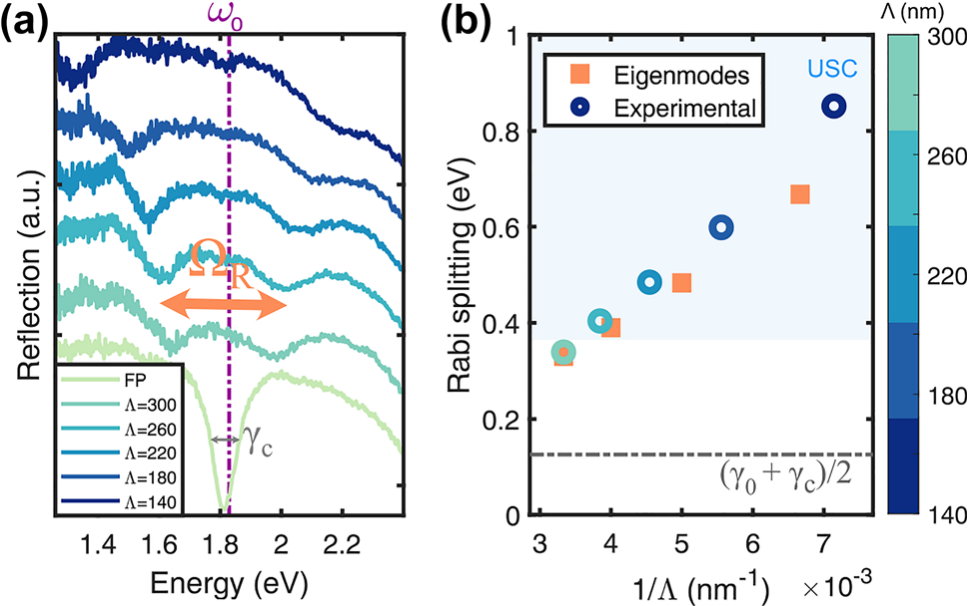
Rabi splitting scaling with the pitch at zero detuning. (a) Experimental reflection at near-normal incidence of an empty FP microcavity and with nanodisks of *d* = 80 nm (at zero detuning) for different pitches (variation in color). The Rabi splitting (orange arrow) decreases with the pitch. (b) Linear dependence of the Rabi splitting and 1/Λ. Comparison between the experimental value obtained as the difference of the minima in reflection and the theoretical eigenfrequencies of the equivalent thin film. Both for *d* = 80 nm, *t* = 30 nm. The shaded area marks the ultrastrong coupling regime.

The modes split after placing the nanodisks inside a resonantly tuned (*δω* = *ω*_*c*_ − *ω*_0_ = 0) microcavity, resulting in two dips in reflection as shown in Figure 3a. The mode splitting at zero detuning in the real frequency is called *Rabi* splitting,

(2) $$\Omega_{R}=\omega_{+}{|}_{\delta \omega =0}-\omega_{-}{|}_{\delta \omega =0}.$$

To quantify the Rabi splitting, we assume that it is given by the energy difference between the two reflection dips at zero detuning, as shown in Figure 3a for various pitches. This procedure may suffer from inaccuracies, as demonstrated previously [46, 47]. Therefore, we compared the resulting values of Rabi splitting obtained from measured spectra (circles) to that from the pole-search method for the equivalent thin film (squares) in Figure 3b. The agreement is good for diluted arrays but deteriorates for denser arrays. This is mainly due to the UP approaching the onset of IBTs in gold, complicating the determination of the experimental reflection minima. Hence, in this spectral range, the pole-search method is more accurate for evaluating the Rabi splitting. Moreover, note that the Rabi splitting in Figure 3b exhibits a linear increase inversely proportional to the array pitch, Ω_*R*_ ∝ Λ^−1^ (the same scaling is observed in Figure S9 for nanodisks of other diameters), which agrees with previous studies [24, 31, 48] and can be understood from the scaling of the coupling strength, $g\propto \frac{\mu_{\text{pl}}}{\Lambda }$ , with the number of particles and their transition dipole moments. Thus, the coupling strength increases for larger nanodisks due to the increase of dipole moment, *μ*_pl_ (*f* in the effective Lorentz model). In practice, obtaining the Rabi splitting with the pole-search method allows estimating the coupling strength as $g\approx \sqrt{{\left(\frac{\Omega_{R}}{2}\right)}^{2}+{\left(\frac{\delta \gamma }{4}\right)}^{2}}$ . Furthermore, in most cases, the difference in uncoupled decay rates is typically small in comparison to the Rabi splitting, leading to Ω_*R*_ ≈ 2*g*. These approximations are not universal but hold fairly well in the studied parameter range, including the onset of USC, see Figure S7 and the corresponding discussion.

**Figure 4:**
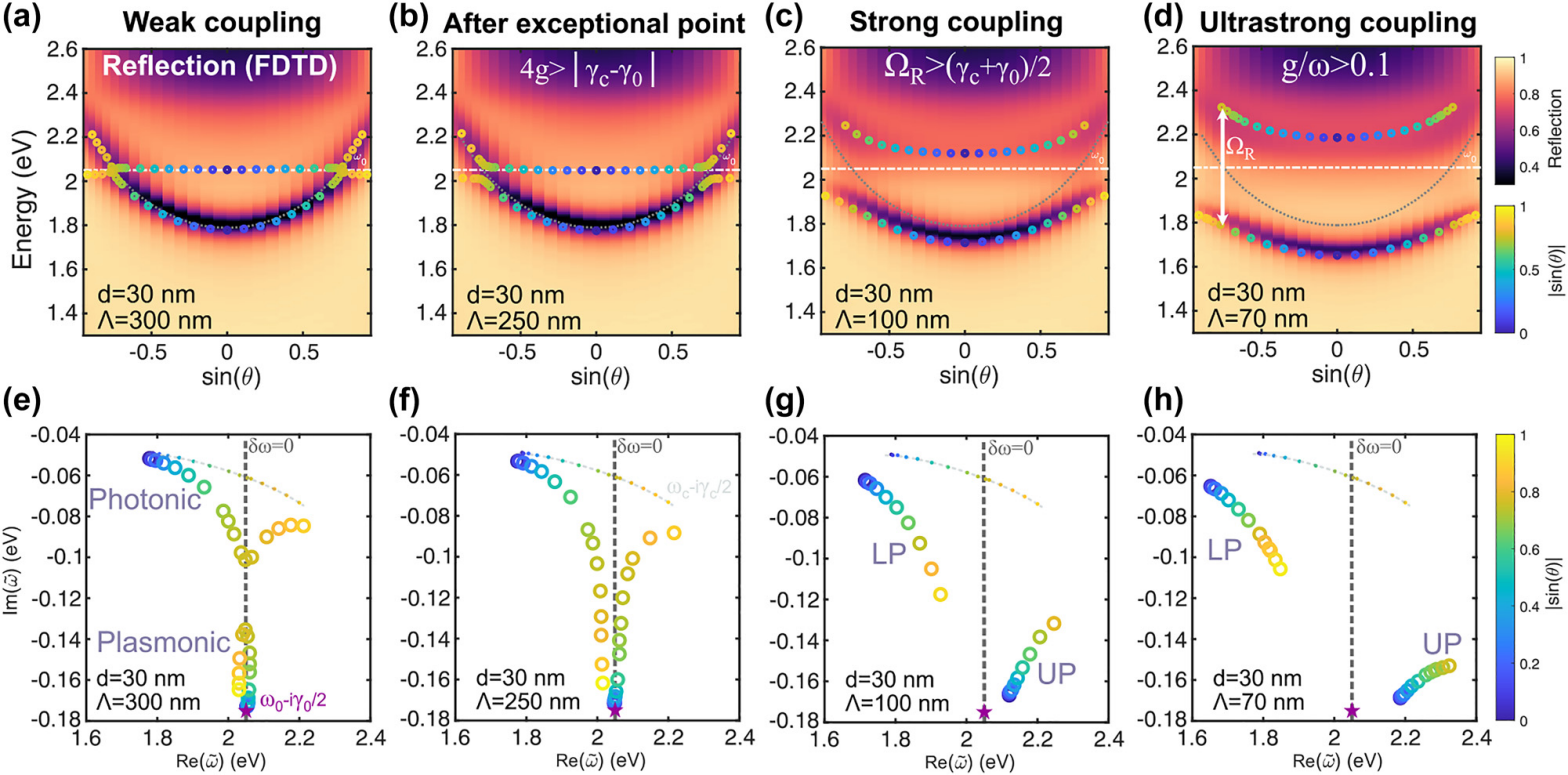
Transition from weak to ultrastrong coupling regime. *Top row:* Angular dispersion in reflection (colormap) of the plasmon–microcavity system calculated by FDTD with the real part of the eigenfrequencies (calculated with the equivalent thin film approximation) in the markers on top. All nanodisks have *d* = 30 nm and *h* = 15 nm, while the microcavity has *t* = 40 nm and *L* = 180 nm. The coupling strength between them varies with the pitch, Λ, covering several interaction regimes: (a) weak coupling, (b) beyond the exceptional point, 4*g* > |*γ*_*c*_ − *γ*_0_|, (c) strong coupling, and (d) ultrastrong coupling. *Bottom row:* Eigenfrequencies in the complex-frequency plane for various angles (colorbar) of the coupled system with the same equivalent thin film. (e) In weak coupling, there is a clear distinction between the photonic (close to the dash-dotted curve) and plasmonic modes (close to the purple star). (f) Beyond the exceptional point, the trajectories split in the real frequency plane. (g) In strong coupling, the Rabi splitting overcomes the average uncoupled decay rate. (h) Ultrastrong coupling: a clear polaritonic gap separates both polaritonic branches. The dispersive bare cavity mode, *ω*_*c*_ − i*γ*_*c*_/2, is plotted in the dash-dotted curve, while the bare plasmon, *ω*_0_ − i*γ*_0_/2, is marked as a purple star. The gray dashed line marks zero detuning, *δω* = 0.

For a fixed diameter (*d* = 30 nm) and microcavity (*L* = 180 nm), the coupling regime can be tuned from weak to ultrastrong by decreasing the arrays’ pitch from Λ = 300 nm to 70 nm (increasing density). The transition is shown in angle-dependent reflection spectra calculated using FDTD in Figure 4a–d (top panels) that highlight the crossing or anticrossing of the modes depending on the regime. This is particularly visible in the real part of the equivalent thin film eigenfrequencies (circles on top). Note that the coupling strength remains constant for all angles because the dipole moments of nanoparticles are always aligned with the incoming field for *s*-polarization.

Plotting the eigenfrequencies in the complex-frequency plane provides simultaneous information about real frequencies and decay rates as shown in the bottom row of Figure 4. Specifically, in the weak coupling regime, the real parts of the eigenfrequencies closely follow the uncoupled components, marked in a star and a dot-dashed curve in Figure 4e. They become identical at zero detuning (gray dashed line). On the contrary, their imaginary parts approach each other without crossing at zero detuning, as shown by the clear gap between the two QNM branches in Figure 4e along the dashed line.

As the coupling strength increases, the decay rates become even more similar until reaching the exceptional point (EP), where both the decay rates and real eigenfrequencies are identical at zero detuning. This occurs when *g* = |*γ*_*c*_ − *γ*_0_|/4 = |*δγ*|/4. The position of the EP is sensitive to *δγ*, which varies with the mirror thickness or its material quality [49]. This means that the same nanodisk array placed in a cavity with thin gold mirrors (lossy cavity) may be found above the EP while being below the EP when placed in a cavity with thicker mirrors as shown in Figure S4.

Further increase of the coupling strength beyond the EP results in a topology change of the eigenfrequencies’ trajectories. They split into two disconnected branches on the real axis. Thus, the degeneracy in real frequency is lifted. This effect is illustrated in Figure 4f. Moreover, Figure 4g and h shows that this topology persists when reaching strong coupling and USC. This is the well-known anticrossing in real frequency dispersion, as depicted in Figure 4c and d where the Rabi splitting is marked with a white arrow. The definition of strong coupling involves the observation of two distinct reflection dips, for which the Rabi splitting must overcome the average decay rates, Ω_*R*_ > (*γ*_*c*_ + *γ*_0_)/2 [4]. We consider this the onset of the strong coupling regime, although two distinct dips are observed even for lower nanoparticle densities, as shown in Figures S5 and S6.

Note that the behavior of the two new branches is not symmetrical in the complex-frequency plane. Moreover, Figure 4h shows that the asymmetry becomes greater with higher coupling strengths. The decay rate of the LP decreases while the decay rate of the UP increases, since $\gamma_{\pm }=2\left|I,m,\left({\tilde{\omega }}_{\pm }\right)\right|$ . This effect has been observed previously in various platforms [6, 12, 13, 31] and will be further explored below. Finally, we note that the eigenfrequencies of the plasmon–microcavity polaritons in the complex-frequency plane display similar behavior to the cavity-free polaritons described previously in Ref. [50].

### 2.4 Asymmetric lower and upper polariton decay rates at zero detuning

**Figure 5:**
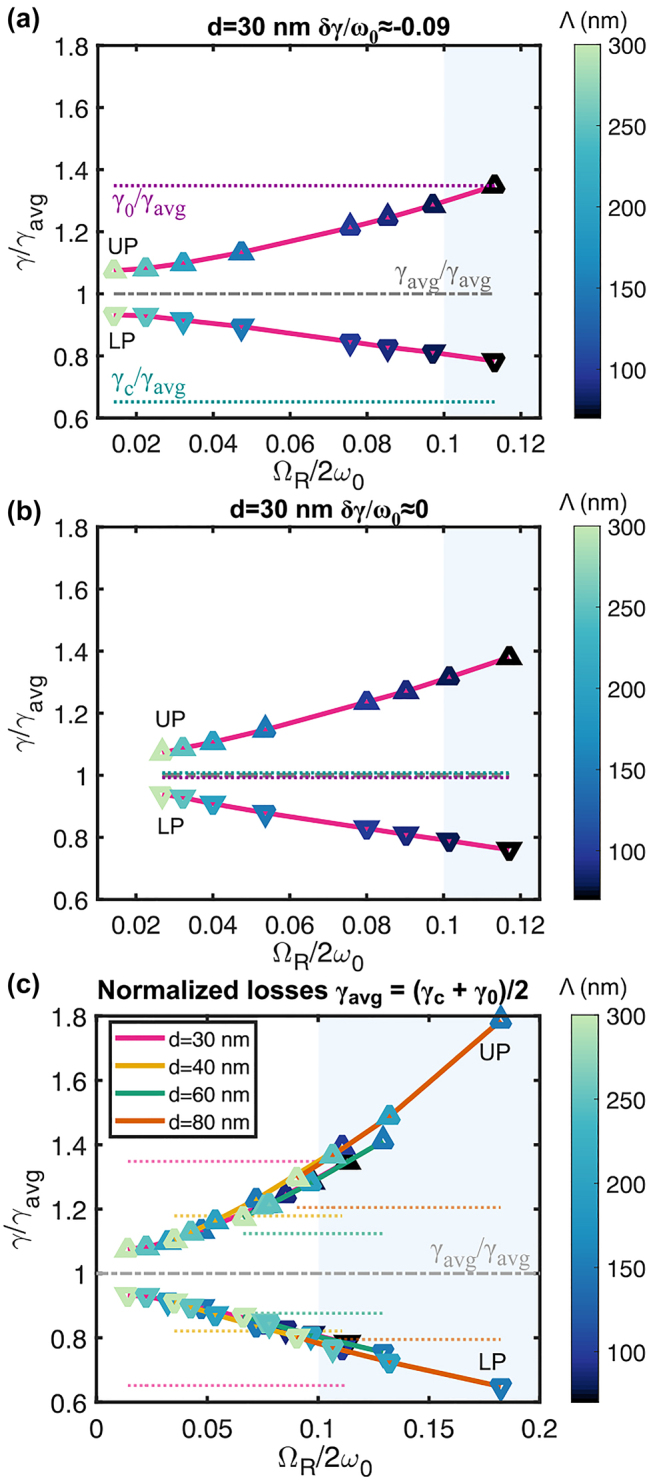
Asymmetry of calculated polaritonic decay rates at zero detuning. (a) Calculated polaritonic decay rates *γ*_±_ normalized by the average decay rate, *γ*_avg_ ≡ (*γ*_*c*_ + *γ*_0_)/2, for thin films equivalent to meta-atoms with *d* = 30 nm coupled to a cavity of *L* = 180 nm and Drude–Lorentz mirrors of *t* = 30 nm, with *δγ* > 0. The uncoupled decay rates are marked in dotted lines. The purple dashed line shows the decay rate of the plasmons outside of the cavity coupled to free space. (b) Same equivalent thin film coupled to a cavity such that *δγ* ≈ 0. The cavity has *L* = 140 nm with mirrors of thickness *t* = 26 nm. (c) *γ*_±_/*γ*_avg_ of all studied equivalent thin films for *d* = 30, 40, 60, 80 nm meta-atoms coupled to the same cavity in (a).

First, let us examine the asymmetry in the decay rates at zero detuning. At *δω* = 0, both polaritons have the same rate, *γ*_±_ = *γ*_avg_ ≡ (*γ*_0_ + *γ*_*c*_)/2 within the coupled oscillators model [4, 5]. Within this model, an asymmetry is introduced only at *δω* ≠ 0. The larger the detuning, the closer the polaritonic decay rates get to those of the uncoupled ones, as in Figure S12 [5]. Nevertheless, even at zero detuning and low normalized coupling strengths (i.e., far from USC), our data in Figure 5a show that the polaritonic decay rates deviate from *γ*_avg_. As the coupling strength increases, the decay rate of the UP rises and approaches the nonradiative decay rate of the effective medium, *γ*_0_. In contrast, the decay rate of the LP decreases and approaches that of the cavity, *γ*_*c*_.

A more dramatic asymmetry with *γ*_+_ > *γ*_0,__*c*_ > *γ*_−_ is found when the cavity decay rate matches that of the equivalent thin film, *δγ* = *γ*_*c*_ − *γ*_0_ ≈ 0 as illustrated in Figure 5b. Matching of the decay rates was achieved by reducing the cavity mirrors thickness, and the cavity thickness was set to *L* = 140 nm to ensure the zero detuning condition. Once more, the decay rate of the LP (UP) decreases (increases) as the coupling strength increases, such that the LP reaches even 75 % of *γ*_0_. As discussed before, this observation goes beyond the coupled oscillator model.

Reaching *γ*_+_ > *γ*_0,*c*_ > *γ*_−_ is also possible with unequal uncoupled decay rates, *δγ* ≠ 0. Figure 5c shows the normalized decay rates for all the equivalent thin films studied here, with their uncoupled losses in dotted lines in the background. Each diameter has a different *δγ*, and for most of them, the coupling strength is sufficiently high to achieve *γ*_+_ > *γ*_0,*c*_ > *γ*_−_. Moreover, they all follow the same pattern indicating that regardless of *δγ*, if the normalized coupling strength is high enough, the decay rates of the polaritons will pass over the boundaries of the uncoupled constituents, such that *γ*_+_ > *γ*_0,*c*_ > *γ*_−_ is eventually reached. These observations have been experimentally verified in previous studies [20, 26, 31], reaching even situations where a polaritonic photoluminescence linewidth was one order of magnitude narrower than the uncoupled excitons [13].

In contrast to previous studies [6, 7, 11], a recent publication by Wang et al. has shown that polaritonic decay rate asymmetry can occur even in systems with negligible disorder [12], which is the case in our study. They attributed the decay rate imbalance to incoherent energy transfer between uncoupled components [12]. Their model explains the asymmetry for zero detuning and predicts that for *δγ* ≈ 0 the polaritonic decay rates can go beyond the uncoupled ones, as observed in our data in Figure 5b. Within that model, the decay rates increase (or decrease) at the same rate with increasing the coupling strength. However, in Figure 5c, the UP increase is larger than the decrease in LP. This occurs because the UP is spectrally closer to the IBTs onset. The higher the coupling strength the closer the UP is to the IBTs, leading to higher decay rates as observed in Figure 5c, and resulting in an increase of the total decay rate as shown in Figure S9. Replacing the mirrors’ response with an artificial pure Drude model (no IBTs component) shows that the rates of growth and reduction are practically the same as shown in Figure S10d.

As discussed before, the average uncoupled decay rates considered for the normalization in Figure 5a–c contain the uncoupled intrinsic plasmonic decay rate, *γ*_avg_ = (*γ*_*c*_ + *γ*_0_)/2, because the cavity shields the extrinsic (mainly radiative) losses. However, using the FWHM of the plasmonic response in free space, *γ*_pl_, may be tempting because it is readily available from reflection measurements. The behavior of the decay rates in that case is discussed in Figure S11.

### 2.5 Asymmetric polaritonic decay rates at nonzero detuning

**Figure 6:**
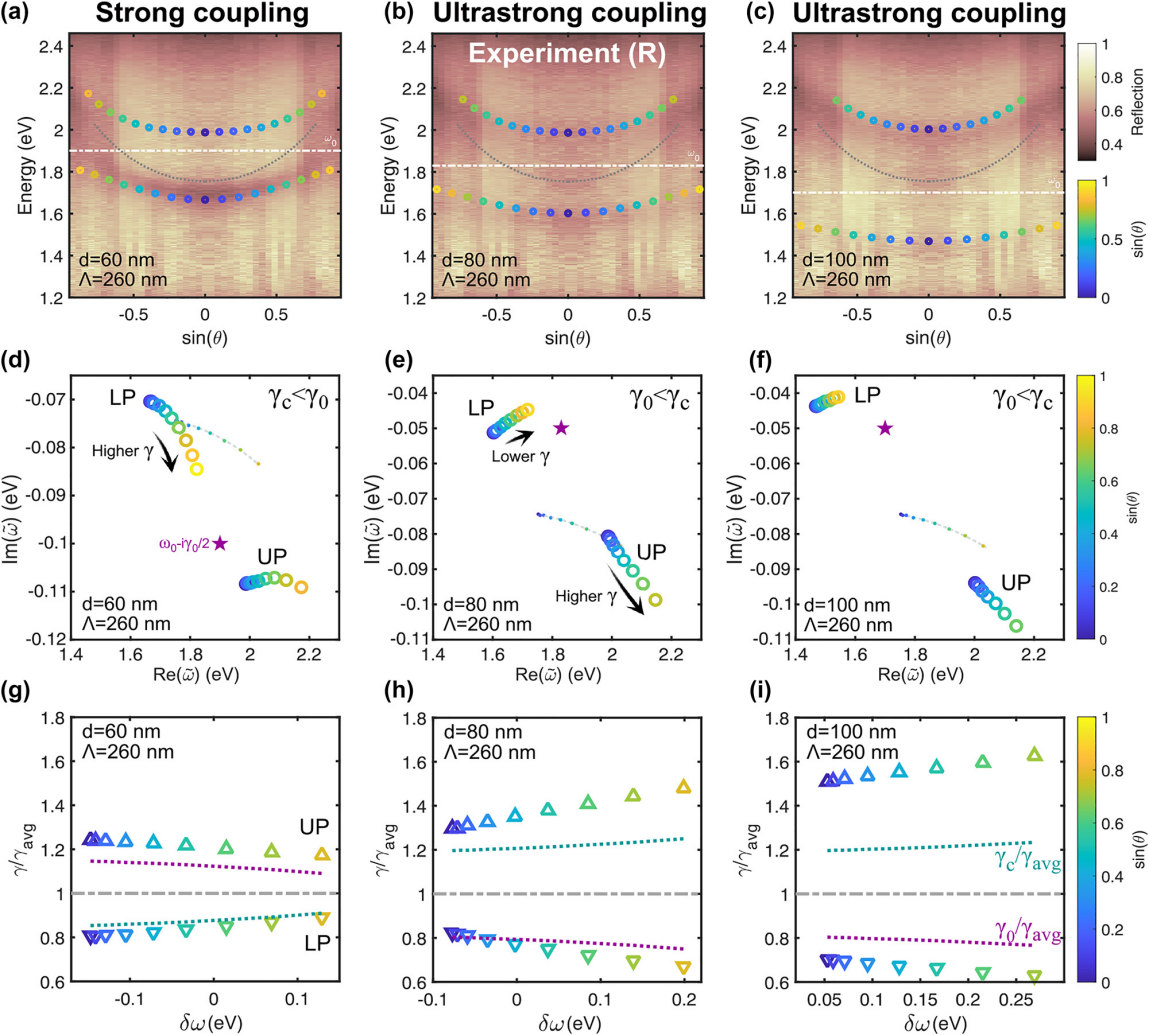
Polaritonic decay rates variation with detuning, *δω* ≠ 0. *Top row:* Measured angle-dependent reflection spectra of nanodisk-microcavity structures with various nanodisk diameters for fixed Λ = 260 nm, *t* = 30 nm, and *L* = 180 nm. The real eigenfrequencies of the QNMs are plotted on top in dots. (a) *d* = 60 nm with Ω_*R*_/2*ω*_0_ = 0.08. (b) *d* = 80 nm with Ω_*R*_/2*ω*_0_ = 0.1. (c) *d* = 100 nm with Ω_*R*_/2*ω*_0_ = 0.16. *Middle row:* (d–f) Eigen- frequencies in the complex-frequency plane with the same parameters as above. The purple stars correspond to the bare uncoupled plasmon, *ω*_0_ − i*γ*_0_/2. The dotted lines mark the empty cavity dispersion. *Bottom row:* Normalized decay rates variation with detuning extracted from the plots above. The turquoise (purple) dashed lines mark the normalized decay rates of the uncoupled cavity (plasmon).

Now, let us explore the impact of varying the detuning on the decay rates. To illustrate this, we conducted dispersion in reflection measurements using nanodisk arrays of different diameters while keeping the cavity thickness constant, as shown in Figure 6. The pitch is fixed to 260 nm, so the coupling strength increases only with the nanodisk diameter. The top row shows that the dips in experimental reflectivity correspond to the eigenfrequencies plotted on top. As mentioned earlier, the slight mismatch observed in Figure 6c is due to the IBTs.

The left column in Figure 6 displays the scenario when the decay rate of the bare plasmon is higher than the cavity, *γ*_0_ > *γ*_*c*_. In this case, the trajectories resemble those in Figure 4, where the decay rate of the LP increases as it approaches the bare plasmon in the complex-frequency plane, *γ*_−_ → *γ*_0_. However, in this case, the polaritonic decay rates are not bounded by the uncoupled ones, *γ*_−_ < *γ*_*c*,0_ < *γ*_+_.

The opposite case, where the decay rate of the cavity is larger than the bare plasmon, *γ*_0_ < *γ*_*c*_, is illustrated in the middle and right columns in Figure 6. The diameter increase leads to USC for both cases, causing unbounded polaritonic decay rates and flattened dispersion, particularly for Figure 6c. This results in a relatively narrow LP with a flat frequency dispersion, which may find applications in refractive-index sensing.

The bottom row in Figure 6 shows that the decay rates of the polaritons are not equal for *any* detuning. This is different from the cross-damping model discussed previously, where the decay rates of the polaritons were found to be the same as the average loss for a higher detuning [12]. This difference is due to the additional loss caused by the IBTs. If we artificially remove them, we can see that both polaritons have the same loss when *δ*ω ≠ 0, as shown in Figure S13.

### 2.6 Decay rate asymmetry in bulk polaritons

The polaritonic linewidth asymmetry found in our plasmon–microcavity systems is present even when plasmonic nanoparticles are approximated with an effective Lorentz permittivity, suggesting it to be a generic phenomenon. To verify the limits of this generality, it is instructive to examine how the observed effect relates to other nanophotonic systems. Purely plasmonic systems consisting of several interacting plasmonic nanoparticles are well-known to exhibit asymmetric linewidths in their spectroscopic response. Specifically, such asymmetric linewidth states are referred to as superradiant (broad) and subradiant (narrow) states, and they arise due to symmetric and antisymmetric combinations of plasmonic excitations in individual nanoparticles comprising the interacting system [51–53]. In this sense, these states are similar to the upper and lower polaritons discussed in this work, which arise due to symmetric and antisymmetric combinations of plasmonic and cavity excitations. Furthermore, due to the significant differences in the linewidths of the superradiant and subradiant states, they often exhibit asymmetric spectral lineshapes as a result of Fano interference [51–53]. These plasmonic nanostructures typically deal with finite-size objects and involve far-field radiation interference in a specific scattering channel. Below, we consider a situation of an infinite bulk polariton system [54] and show that despite the absence of any scattering channels, this system exhibits polaritonic linewidth asymmetry.

**Figure 7:**
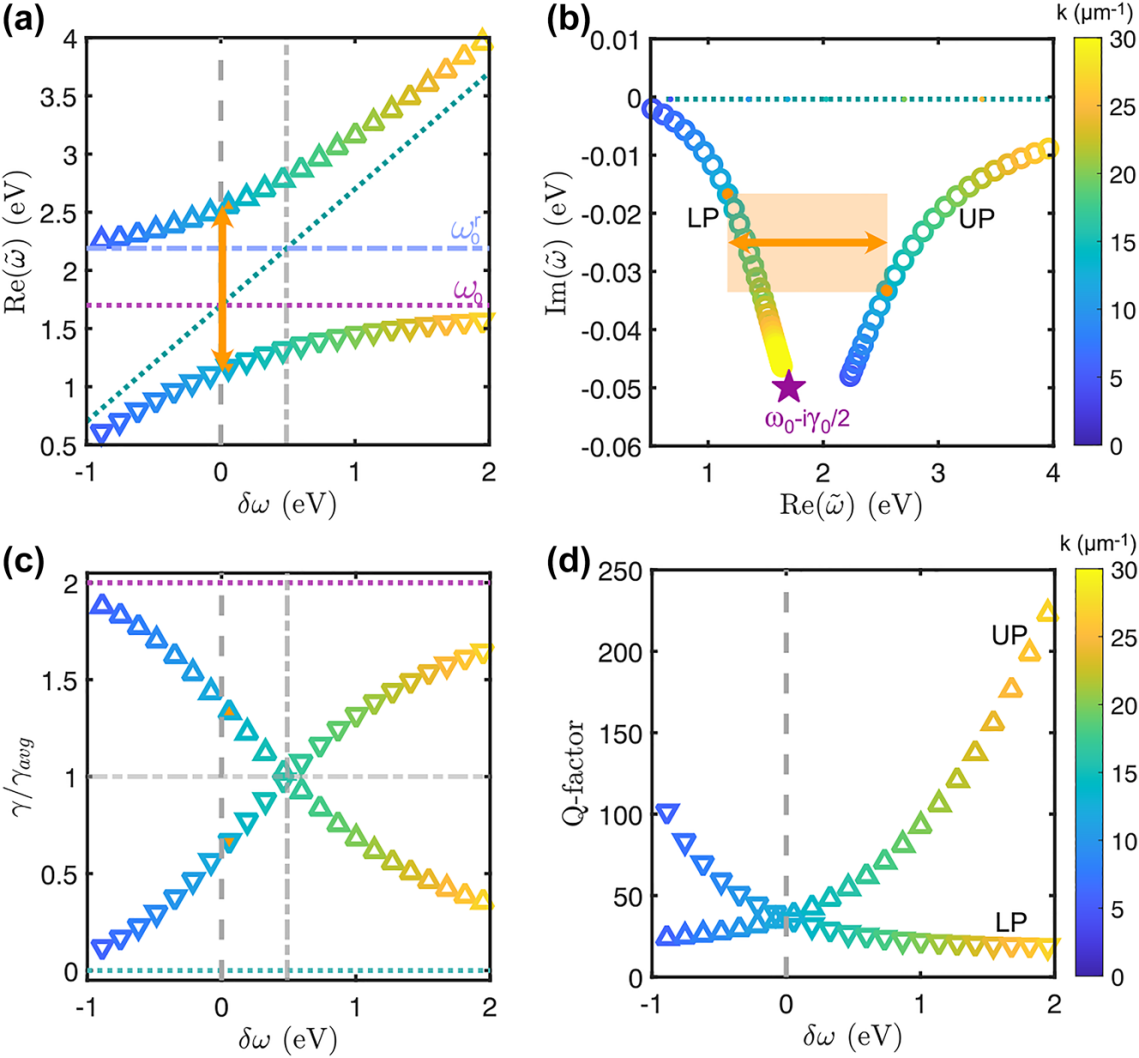
Decay rates asymmetry in bulk polaritons. (a) Real eigenfrequencies of the bulk material with an effective permittivity of the equivalent thin film of an array of *d* = 100 nm and Λ = 260 nm. The detuning is considered with respect to the plane wave in the material without resonances (turquoise dotted line). The Rabi splitting is marked with an orange arrow. The renormalized frequency is in a blue dash-dotted line, $\omega_{0}^{r}=\sqrt{\omega_{0}^{2}+4g_{B}^{2}}$ , and its corresponding detuning with the light line is the gray dash-dotted line. (b) Polaritonic eigenfrequencies of the bulk material in the complex-frequency plane. A shaded orange square connects the eigenfrequencies at zero detuning such that its width (orange arrow) is the Rabi splitting. The UP and LP have different imaginary parts that translate into decay rates. (c) Normalized decay rates for the polaritons with varying detuning. An orange filling highlights the asymmetry of the decay rates at zero detuning (vertical dashed line). The uncoupled decay rates are marked in dotted lines. A vertical dash-dotted line marks the detuning corresponding to the renormalized frequency, $\omega_{0}^{r}$ . (d) Quality factor, $Q=\frac{\omega }{\gamma }$ , of the bulk polaritonic modes. The UP and LP share the same quality factor at zero detuning.

A bulk polariton is a normal mode of resonant bulk material, as demonstrated by Hopfield [54]. Bulk polaritons do not require a microcavity; instead, the material resonance hybridizes with a plane wave traveling through the material. These modes are found as roots of its dispersion equation, $kc-\omega \sqrt{\varepsilon \left(\omega \right)}=0$ , where *k* is the free-space wavevector. Figure 7 shows the bulk polaritons of a material with the effective permittivity of an equivalent thin film corresponding to nanodisks of *d* = 100 and Λ = 260 nm. The corresponding microcavity polaritons are shown in Figure 6c. Bulk polaritons display a well-known anticrossing in the real part of the eigenfrequencies as shown in Figure 7a by varying the detuning between the plane wave and the material resonance. The orange arrow marks the *bulk* Rabi splitting, $2g_{B}=\omega_{P}\sqrt{f{/\varepsilon }_{\infty }}$ , which bounds the coupling strengths of the material with any optical modes [50].

In Figure 7b, it can be observed that the eigenfrequencies in the complex-frequency plane are similar to the microcavity polaritons discussed in Figure 4. Even at zero detuning (marked in orange), the trajectories are asymmetric. The decay rate asymmetry is even more apparent in Figure 7c, where the UP is larger than the LP at zero detuning (dashed line). Here, *γ*_avg_ = *γ*_0_/2 because *γ*_*c*_ = 0 due to the absence of a cavity. At higher detuning, *δ* ≈ 0.46 eV, marked in a gray dot-dashed line, the decay rates become equal *γ*_±_ = *γ*_avg_. This detuning corresponds to the renormalized resonance frequency $\omega_{0}^{r}=\sqrt{\omega_{0}^{2}+4g_{B}^{2}}$ [17, 23], which is marked with a blue dot-dashed line in Figure 7a. This frequency limits the upper polariton, opening the polaritonic band gap, $\Delta =\omega_{0}^{r}-\omega_{0}$ [50]. A similar behavior is found for the plasmon–microcavity system with Drude mirrors for the same equivalent thin film presented in Figure S13i. However, the detuning at which the decay rates are equal does not correspond to the renormalized resonance frequency, $\omega_{0}^{r}$ , because of the inclusion of additional losses by the cavity. The observation of asymmetry in the linewidth of bulk polaritons suggests that this phenomenon is more general than initially assumed. At the same time, this behavior should be possible to trace using the Hopfield Hamiltonian with losses [54] and corresponding effective coupled oscillator models [55]. The latter, however, goes outside the scope of this study.

Interestingly, despite the asymmetry in decay rates, Figure 7d shows that the quality factors of polaritonic eigenstates, calculated as *Q* = *ω*/*γ*, match at zero detuning. The balance of the quality factors can be physically interpreted as both polaritons having the same number of oscillations before damping. As the coupling strength increases, the polaritonic eigenfrequencies diverge further, causing an increase in linewidth asymmetry to ensure the balance in quality factors. This is important because bulk polaritons sustain the maximum Rabi splitting that can be reached for a given material [16, 50]. Thus, they will also show the largest linewidth asymmetry that can be reached if no additional losses are introduced to the system via the cavity. Note that this is not the case with our plasmon–microcavity hybrid, where the IBTs in the mirrors enhance the asymmetry. In that case, a large Rabi splitting causes the UP to spectrally overlap with the IBTs, increasing its decay rate. Therefore, the IBTs hinder the balancing of the polaritonic quality factors at zero detuning as shown in Figure S15.

## 3 Conclusions

This study investigated the decay rates of plasmonic meta-atom–microcavity systems in different light–matter interaction regimes as a model for polaritons. We opted for an entirely classical approach to avoid the limitations of the commonly used non-Hermitian Hamiltonians in single-mode and rotating-wave approximations. To do so, we numerically simulated the system’s reflection using FDTD and measured it experimentally. Then, we used these data to approximate the reflection of the plasmonic nanodisk array to that of an effective homogeneous film with a Lorentzian permittivity. This allowed us to find the eigenfrequencies and decay rates using the pole-search approach.

Our findings reveal that the interactions with the continuum of free-space modes increase the overall decay rate of the meta-atoms outside the cavity due to increased radiative losses. However, when the system is coupled, the microcavity protects against this broadening. Tracking the eigenfrequencies of the coupled system in the complex-frequency plane for systems in different interaction regimes shows that a change in the topology of eigenfrequency trajectories can be observed upon reaching the exceptional point. Beyond the exceptional point, their topology remains intact, even after transitioning to the strong and ultrastrong coupling regimes. By studying the real and imaginary parts of the eigenfrequencies separately, we could monitor the dispersion and decay rates, respectively.

Our analysis indicates that the polaritonic decay rates deviate from the average of the uncoupled rates, contrary to the predictions of effective coupled oscillator models, but consistent with previous experiments on exciton–polaritons [6, 12, 56]. Moreover, even though our system neglects disorder, it consistently shows an asymmetry in the polaritonic linewidths at zero detuning. This is observed in the entire studied parameter range. Even more so, for systems with high coupling strengths or similar cavity and plasmon decay rates, the polaritonic decay rates surpassed the uncoupled ones such that *γ*_−_ < *γ*_*c*,0_ < *γ*_+_. This suggests that ultrastrong coupling between lossy plasmons and lossy microcavities can result in a narrow polariton, which could be useful for, e.g., sensing applications. This concept may be generalized for other materials with high decay rates.

Our study suggests that linewidth asymmetry is a classical electromagnetism phenomenon, which does not require the knowledge of details about the microscopic processes determining its linewidth. Moreover, the linewidth asymmetry is present even for bulk polaritons. However, we noted that the quality factors of bulk polaritons match at zero detuning. Therefore, in the absence of additional losses, the asymmetry in the decay rates arises to balance the large difference in the polaritonic frequencies given by the Rabi splitting.

## 4 Methods

### 4.1 Sample preparation

The structures were fabricated on 170 μm glass coverslips (Deckglaser #1.5). The substrates were cleaned in acetone and isopropanol at 50 °C in ultrasonication, and then N_2_ blow-dried and cleaned with oxygen plasma. The bottom mirror was deposited (30 nm of gold with 2 nm of Cr for adhesion) by using an electron beam evaporator (Kurt J. Lesker PVD225). Then, SiO_2_ was deposited using STS plasma-enhanced chemical vapor deposition (PECVD) at 300 °C. Each sample had SiO_2_ thickness, accounting for half of the total thickness of the microcavity (80 nm). The 40 × 40 μm nanodisk arrays were patterned using a standard electron beam-lithography process (Raith EBPG 5200). Arrays with different diameters (*d* = 60, 80, 100 nm) and separated by different pitches (Λ ∈ [140, 340] nm) were patterned using poly(methyl methacrylate) (PMMA) as resist. Note that PMMA and SiO_2_ share similar refractive indices in the visible. The height of the disks was given by the 20 nm of gold that was deposited by e-beam evaporation. After lift-off, the samples were annealed for 10 min at 300 °C to improve the nanodisks’ crystallinity. A PMMA layer of (80 nm) was spin-coated on top of the patterned nanodisks before closing the microcavity by evaporating 30 nm of gold as a top mirror.

The scanning electron microscopy (SEM) images were obtained by coating the nanodisks arrays with a thin layer of conductive polymer (E-spacer) using a Zeiss Supra 60 VP.

### 4.2 Optical measurements

The near-normal incidence was measured using a 20× objective (NA = 0.45, Nikon) and was collected using a multimode fiber optic patch cable (NA = 0.22, 105 μm diameter). Dispersion measurements in reflection were performed by imaging the Fourier plane using an inverted microscope (Nikon Eclipse, TE2000-E). The excitation source was a collimated beam of a halogen lamp that was focused on the sample with a 40× objective (Nikon NA = 0.95 MRD70470). The spectrum of the lamp used to normalize the signal was obtained for each angle with a silver mirror deposited on the same substrate as the sample. The Fourier plane of the objective was imaged with a Bertrand lens. Then, the spectra for different radii in the Fourier plane were collected simultaneously for several angles with a fiber bundle consisting of 19 fibers with 100 μm core (Andor SR-OPT-8002). The fiber bundle was coupled to a spectrometer (Andor Shamrock SR-500i, equipped with a CCD detector Andor Newton 920).

### 4.3 Pole-search approach

The frequency-dependent scattering matrix of the equivalent film of meta-atoms in the basis of right- and left-propagating plane waves reads

(3) $$\hat{S}\left(\omega \right)=\left(\begin{array}{cc} r_{LL}\left(\omega \right) & t_{LR}\left(\omega \right)\\ t_{RL}\left(\omega \right) & r_{RR}\left(\omega \right) \end{array}\right),$$

where *r*_*μμ*_ is the reflection coefficient on side *μ* of the film, and *t*_*μν*_ is the transmission coefficient from *μ* to *ν*. In this case, we considered the glass substrate on the left and the air on the right. Except for the bare meta-atoms, they were fully embedded in glass.

The system is not symmetric because the two scattering channels are embedded in media with different refractive indices. The refractive index of glass and air were considered to be 1.46 and 1, respectively. The reflection and transmission coefficients on both channels, *r*_*L*,*R*_(*ω*) and *t*_*L*,*R*_(*ω*), were calculated with the TMM [57].

Electromagnetic eigenfrequencies $\tilde{\omega }=\omega^{\prime }-\text{i}\gamma$ of the film are then associated with poles of eigenvalues of the $\hat{S}$ -matrix:

(4) $$r_{LL}+r_{RR}\pm \sqrt{{\left(r_{LL}-r_{RR}\right)}^{2}+4t_{LR}t_{RL}}=\infty ,$$

which represents the characteristic equation of the system.

The reflection and transmission coefficients were calculated by TMM [57]. The layers in consideration for the bare nanodisks were: glass/Lorentz/glass. Whereas for the coupled configuration, the following layers were considered: glass/Au/SiO_2_/Lorentz/SiO_2_/Au/air.

The permittivity of the gold mirrors was fitted with a Drude–Lorentz model to the experimental data [38]. The resulting permittivity was:

(5) $$\begin{array}{ll} \varepsilon \left(\omega \right) & =\varepsilon_{\infty }-\frac{\omega_{P}^{2}}{\omega^{2}+\text{i}\gamma \omega }+\frac{f\omega_{P,1}^{2}}{\omega_{0,1}^{2}-\omega^{2}-\text{i}\gamma_{0,1}\omega }+\frac{f\omega_{P,2}^{2}}{\omega_{0,2}^{2}-\omega^{2}-\text{i}\gamma_{0,2}\omega }, \end{array}$$

where *ω*_*P*_ = 8.5 eV, *γ* = 0.045 eV, *ɛ*_∞_ = 2.27 for the Drude term. For the Lorentz part: *ω*_0,1_ = 3 eV, *γ*_0,1_ = 0.9 eV, $f\omega_{P,1}^{2}=7.2$ eV^2^, *ω*_0,2_ = 4.3 eV, *γ*_0,2_ = 2.5 eV, $f\omega_{P,2}^{2}=57.3$ eV^2^. The presence of two Lorentz terms allows describing the IBTs of gold. Therefore, the first two terms in eq. (5) simply give the Drude permittivity when artificially removing the IBTs.

The parameters obtained via fitting with TMM in the Lorentzian permittivity for each nanodisk array are summarized in Table S1.

The eigenfrequencies of the QNMs were obtained as described in the main text by finding the poles of the eigenvalues of the scattering matrix shown in eq. (3).

### 4.4 FDTD simulations

Numerical finite-difference time-domain (FDTD) simulations were performed using commercial software (FDTD solutions, Lumerical, Inc. Canada). Dispersion in reflection spectra was obtained using a linearly polarized incident plane wave source with incident angles such that sin*θ* ∈ [−0.97, 0.97]. The lattice was considered infinite by using periodic boundary conditions with symmetries. The gold permittivity was taken from Johnson & Christy experimental data [38]. SiO_2_ was modeled as a nearly dispersion-free and lossless dielectric with a refractive index of 1.46.

Simulations were obtained for different array pitches (Λ ∈ [70, 360]) and cavity thicknesses (*L* = 160, 170 and 180 nm) for mirrors of *t* = 40 nm. The nanodisk arrays were considered with diameters: 30 nm, 40 nm, 50 nm, 60 nm, 80 nm, and 100 nm with a height was 20 nm for the 3 largest diameters and 15 nm for the 3 smallest to keep the resonance far from the IBTs of gold.

## Supplementary Material

Supplementary Material Details

Supplementary Material Details
