# Supplementary material for: Polaritonic linewidth asymmetry in the strong and ultrastrong coupling regime
Source: Nanophotonics. 2023 Oct 18;12(21):4073–86. doi: 10.1515/nanoph-2023-0492 (PMC11501566; doi:10.1515/nanoph-2023-0492)
Supplement: Supplementary file 2 — Supplementary Material Details [file j_nanoph-2023-0492_suppl_002.pdf]

Supporting Information for
"*Polaritonic linewidth asymmetry in the strong and ultrastrong coupling regime*"

Adriana Canales,[1,*] Therese Karmstrand,[2] Denis G. Baranov,[1,3] Tomasz J. Antosiewicz,[4,1] and Timur O. Shegai[1,†]

[1]*Department of Physics, Chalmers University of Technology, 412 96, Göteborg, Sweden.*

[2]*Department of Microtechnology and Nanoscience (MC2), Chalmers University of Technology, 412 96, Göteborg, Sweden.*

[3]*Center for Photonics and 2D Materials, Moscow Institute of Physics and Technology, Dolgoprudny 141700, Russia.*

[4]*Faculty of Physics, University of Warsaw, Pasteura 5, 02-093, Warsaw, Poland.*

(Dated: September 27, 2023)

CONTENTS

I. Permittivities of equivalent thin films 3

II. Comparison between meta-atom transfer matrix and dispersive effective thin film 4

III. Eigenfrequencies of the equivalent thin film outside of the cavity 6

IV. Exceptional point dependence on the mirror's thickness 7

V. Rabi splitting and coupling strength in plasmon – microcavity systems 10

VI. Increase of total polaritonic decay rates at zero detuning 12

VII. Polaritonic decay rates at zero detuning with Drude mirrors 13

VIII. Asymmetry of polaritonic rates at zero detuning 14

IX. Polaritonic decay rates at non-zero detuning with Drude mirrors 15

X. Quality factors of plasmon – microcavity polaritons 19

References 20

* adriana.canales@chalmers.se

† timurs@chalmers.se

I. PERMITTIVITIES OF EQUIVALENT THIN FILMS

Table S1 summarizes the parameters of the effective permittivities describing the optical response of plasmonic nanodisk arrays. The plots of the permittivities related to the least dense (dashed lines) and densest (solid lines) arrays are shown in Figure S1. The permittivities for other densities will be found between the plotted extremes.

TABLE S1: Values of the Lorentzian fitting of the permittivity of the thin-film approximation for each nanodisk array

Diameter (nm)	ε_∞	ω_0 (eV)	γ_0 (eV)	Pitch (nm)	$f\omega_P^2$ (eV)
30	2.1316	2.05	0.35	70	4.62
				80	3.40
				90	2.67
				100	2.10
				150	0.92
				200	0.51
				250	0.33
				300	0.23
40	2.1316	2.03	0.24	100	4.08
				150	1.75
				200	0.97
				250	0.63
				300	0.44
60	2.1316	1.9	0.2	150	3.61
				200	2.02
				250	1.30
				260	1.22
				300	0.90

				150	6.70
				200	3.45
80	2.1316	1.83	0.1	250	2.21
				260	2.09
				300	1.57
				150	12.70
				200	6.76
100	2.1316	1.7	0.1	250	4.34
				260	4.07
				300	3.03

As explained in the main text, these permittivities are used in the transfer matrix method to further compute the eigenfrequencies of the coupled and bare systems. The thin film used for the calculations has the same height as the nanodisks, which is $h = 15$ nm for $d = 30$ and 40 nm, and $h = 20$ nm for $d = 60, 80, 100$ nm.

II. COMPARISON BETWEEN META-ATOM TRANSFER MATRIX AND DISPERSIVE EFFECTIVE THIN FILM

As mentioned in the main text, Berkhout *et al.* [1] proposed a metasurface transfer matrix for metallic meta-atoms. The transfer matrix accounts for radiation damping and for near- and far-field interactions. They considered an infinitely thin layer with reflectivity and transmissivity ($t_a = 1 + r_a$) related to the plasmonic response of the metasurface. The reflectivity is then

$$r_a(\omega) = \frac{2\pi i k}{\mathrm{A}} \frac{1}{1/\alpha_0(\omega) - \frac{2\pi i k}{\mathrm{A}}} \tag{S1}$$

Where

$$\alpha_0 = \frac{V\omega_0^2}{\omega_0^2 - \omega^2 - i\omega\gamma} \tag{S2}$$

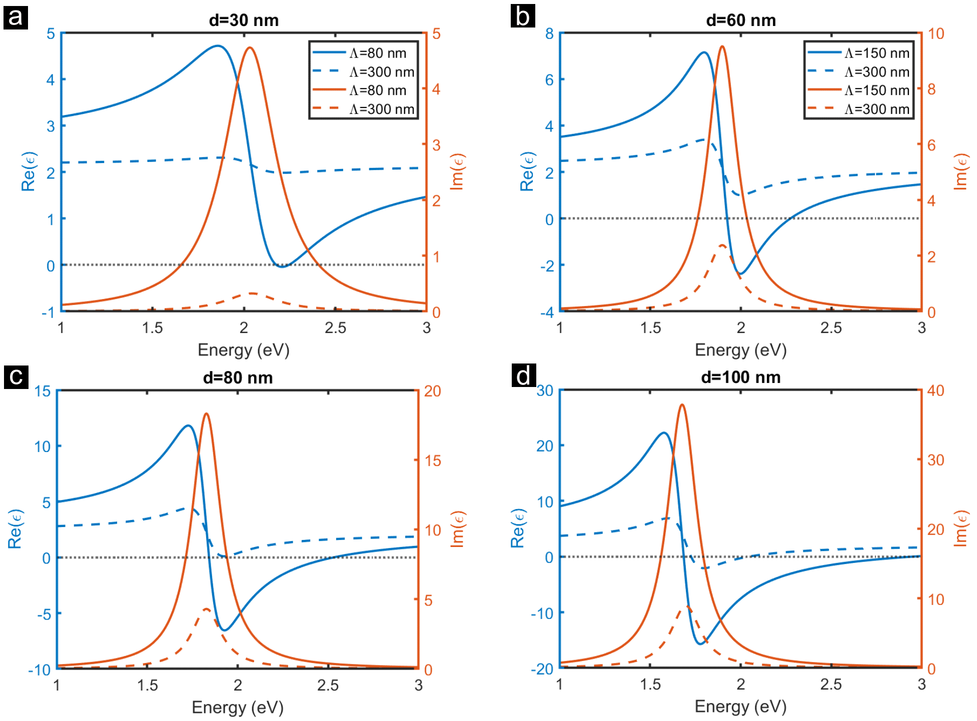

FIG. S1. Effective permittivity of the nanodisk arrays for (a) $d = 30$ nm, (b) $d = 60$ nm, (c) $d = 80$ nm and (d) 100 nm. The solid lines show the permittivity of the densest array considered for each diameter ($\Lambda = 80$ nm for $d = 30$ nm, while $\Lambda = 150$ nm for all the rest) and the dashed lines the permittivity for the less dense one ($\Lambda = 300$ nm for all diameters). The dotted line marks $\Re\varepsilon = 0$.

Here, ω_0 is the resonance frequency and is considered to be the same as in Table S1. γ is the Ohmic decay rate. V is a measure of scattering strength and is proportional to the physical volume, $V_0 = \pi h(d/2)^2$, and $\mathrm{A} = \Lambda^2$ is the unit cell area.

In Figure S2, we show the reflection spectra using the metasurface T-matrix discussed above. Moreover, we compare it to the FDTD calculation, and the Lorentz permittivity of the same system.

First, we compare the reflection of plasmonic nanoparticles embedded in glass in Figure S2a. The Lorentz permittivity and the metasurface T-matrix fit similarly to the FDTD spectra when $V = V_0/3.3$ (dash-dotted lines in Figure S2). However, the mode splitting given by the metasurface T-matrix is not enough to match the FDTD calcula-

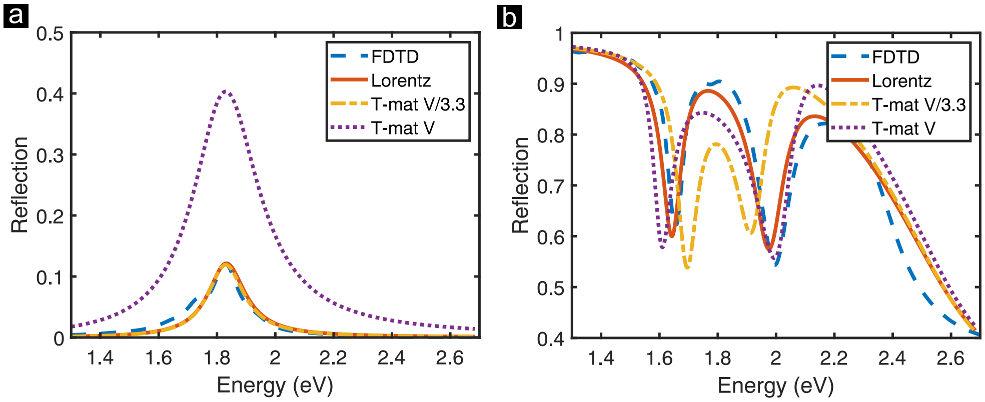

FIG. S2. Comparison between TMM with a metasurface T-matrix and a dispersive equivalent film with a Lorentzian permittivity. (a) Reflection of the plasmonic nanoparticle array and its equivalent thin film embedded in glass. The plasmonic nanodisks arrays have $d = 80$ nm and $\Lambda = 300$ nm for all three methods. (b) Reflection of the meta-atom in a gold microcavity of $t = 40$ nm and $L = 180$ nm. The FDTD calculations are shown in dashed lines. The calculation with a Lorentz permittivity is shown in a solid line. The calculations with the metasurface T-matrix are shown for $V = V_0$ in dotted lines and for $V = V_0/3.3$ in dot-dashed lines.

tions when it is introduced into the microcavity. When considering $V = V_0$, the coupled spectrum fits better, but the corresponding spectrum outside of the microcavity did not match the FDTD calculation, as shown in dotted lines in Figure S2. In the end, using the Lorentz permittivity for a thin film was the best fit for the data inside and outside the microcavity.

III. EIGENFREQUENCIES OF THE EQUIVALENT THIN FILM OUTSIDE OF THE CAVITY

The trajectories of the eigenfrequencies of the bare equivalent thin film embedded in glass were discussed in Figure 2d. Figure S3a includes the other diameters studied here. The discussion from the main manuscript is applicable here as well. In short, the decay rates are given by radiative and non-radiative losses. Radiative coupling to the environment increases the imaginary component of the eigenfrequencies, thus pushing

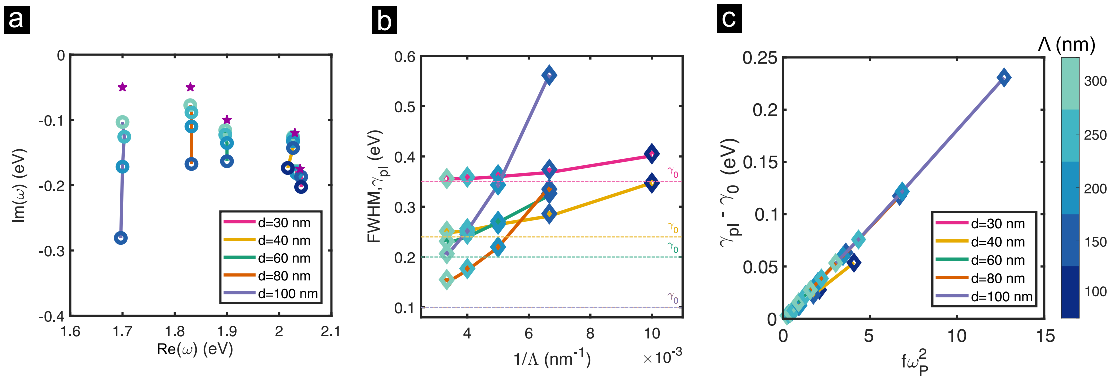

FIG. S3. (a) Trajectories of the eigenfrequencies of the equivalent film in the complex frequency plane for all diameters studied, $d = 30, 40, 60, 80$ and 100 nm. The stars mark the *bare* plasmon, $\omega_0 - i\gamma_0/2$. The pitch, Λ, gives the colormap on the right. The colors corresponding to each diameter are consistent throughout the manuscript. (b) The resonant FWHM of the equivalent films obtained by fitting the reflection spectra for all pitches and nanodisk diameters is shown in curves colored according to the diameter. The decay rates of the thin film QNMs, γ_{pl}, are shown in diamond markers with a colormap given by the pitch. The dashed lines indicate the corresponding non-radiative decay rates γ_0. (c) Linear dependence of the radiative decay rate, $\gamma_{pl} - \gamma_0$, with the oscillator strength ($f\omega_P^2$) of the equivalent thin-film of the meta-atom.

them down in the complex frequency plane. Nanodisks with larger diameters and denser arrays exhibit greater coupling to the environment, leading to higher growth of their imaginary parts relative to the bare plasmon location, $\omega_0 - i\gamma_0/2$. Figure S3b illustrates the increase of the decay rate with respect to the non-radiative component γ_0. Furthermore, we demonstrate that the FWHM of the reflection spectra corresponds to the decay rate of the QNM of the thin film.

IV. EXCEPTIONAL POINT DEPENDENCE ON THE MIRROR'S THICKNESS

Interestingly, as Figure S4 shows, a plasmon – microcavity system composed of the same plasmonic nanodisk array may be found below the exceptional point (EP) or above it, depending on the properties of the Au mirrors. Specifically, when coupled to Au

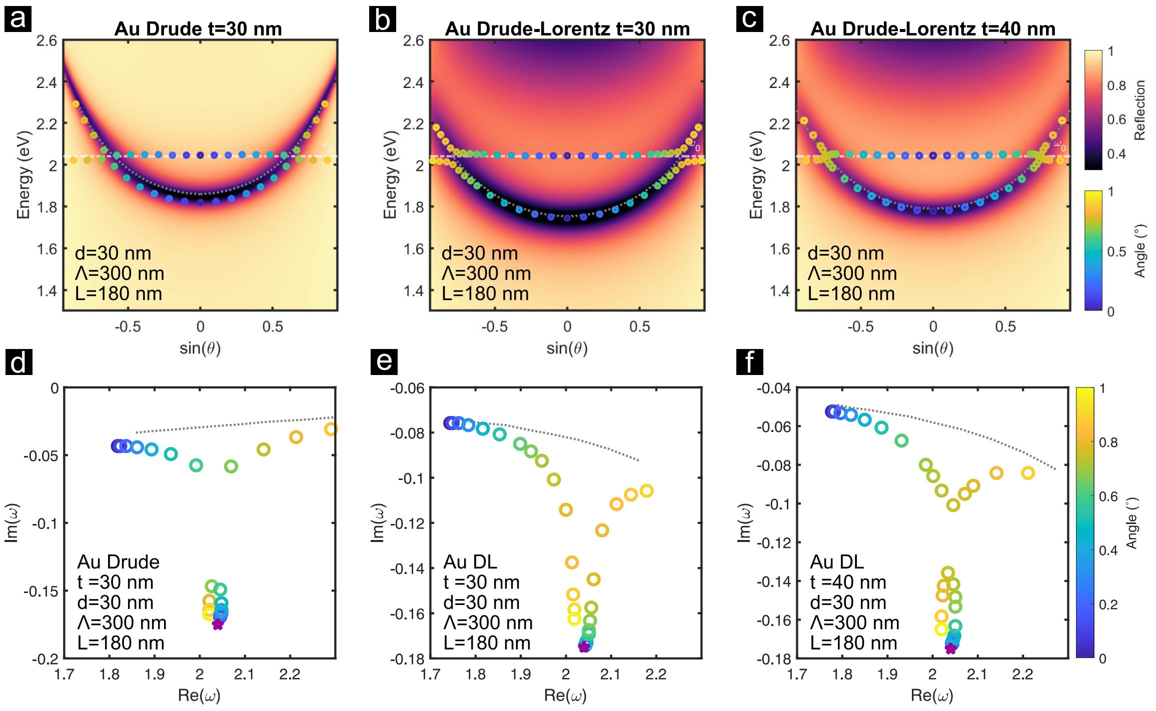

FIG. S4. Sensitivity of the exceptional point to the cavity decay rate. The same array ($d = 30$ nm, $\Lambda = 300$ nm) approximated as a thin film is coupled to similar cavities: (a,d) $t = 30$ nm with Au Drude mirrors (no IBTs), (b,e) $t = 30$ nm with Au Drude-Lorentz (DL) mirrors and (c,f) $t = 40$ nm with Au Drude-Lorentz mirrors.

mirrors described by either Drude permittivity (without interband transitions, IBTs) or Drude-Lorentz permittivity (with IBTs), the system is below or above EP, respectively. This is shown in the first two columns of Figure S4. Although eliminating IBTs in Au is only possible in a thought (numerical) experiment, a qualitatively similar outcome is possible by changing the thickness of the mirrors. As depicted in Figure S4e-f, a plasmonic array coupled to 30 nm Au mirrors operates above the EP, but when coupled to 40 nm ones it is below the EP.

For a better view of the spectra in different regimes, Figure S6 shows a cross-section of the colormaps presented in Figure 3 and Figure S5 for the case of zero detuning.

Note that despite the appearance of splitting in the eigenfrequencies from $\Lambda = 250$ nm, the two polaritonic peaks are not clear until $\Lambda = 150$ nm. However, the strong coupling

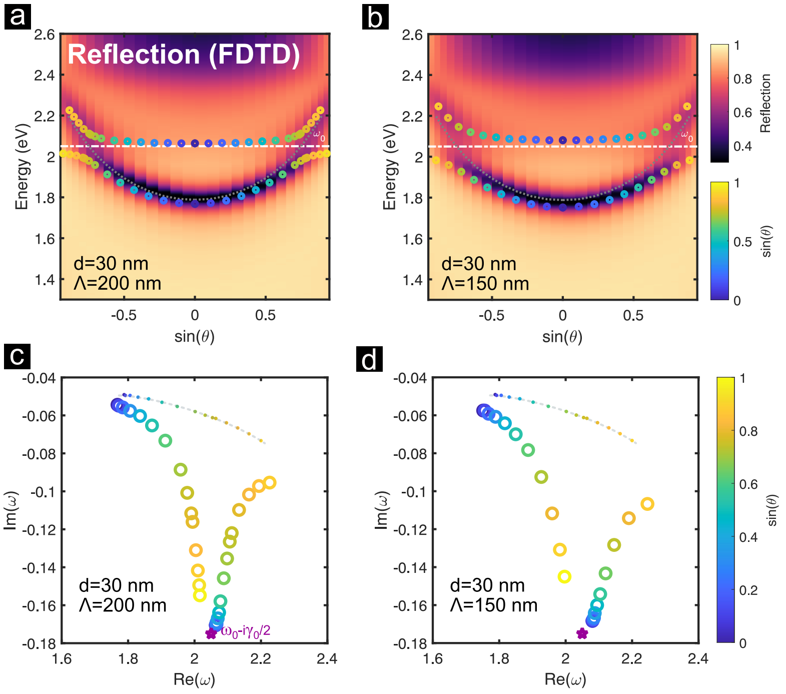

FIG. S5. Reflection (top) and QNMs in complex frequency plane (bottom) of arrays in the regime passed the exceptional point but before the onset of strong coupling. As in figure 3 in the main text: $d = 30$ nm, $t = 40$ nm, $L = 180$ nm with (a, c) $\Lambda = 200$ nm and (b, d) $\Lambda = 150$ nm.

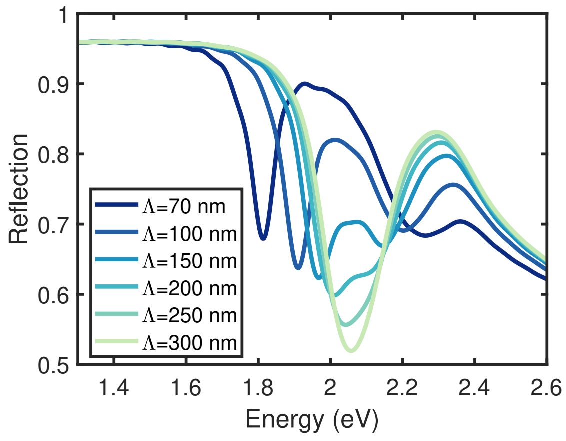

FIG. S6. FDTD simulation of reflection at zero detuning for the microcavity-plasmon coupled system, the data for other angles was presented in Figure 3 and above.

condition, $\Omega_R > (\gamma_0 + \gamma_c)/2$, is not fulfilled until $\Lambda = 100$.

V. RABI SPLITTING AND COUPLING STRENGTH IN PLASMON – MICROCAVITY SYSTEMS

Figure 4 in the main text describes the eigenfrequencies behavior in the complex frequency plane when increasing the coupling strength. The coupling strength in this example increases with the reduction in the array's pitch. The systems beyond the exceptional points will have mode splitting, but that won't necessarily imply strong coupling. Figure S5 shows two more examples (pitches) in between the exceptional point and the strong coupling regime onset. We have discussed that the onset is given by the average losses, $\Omega_R > (\gamma_0 + \gamma_c)/2$[2]. Even though that is the limit considered throughout the text, Figure S5b shows already distinct peaks in reflection even before for $\Lambda = 150$ nm.

The Rabi splitting and the decay rates are readily available by the pole-search method. Therefore, using the definition of the Rabi splitting within the coupled oscillator model the coupling strength, g, can be computed as, [3]:

$$\Omega_R = \omega_+ - \omega_- = 2\sqrt{g^2 - \left(\frac{\delta\gamma}{4}\right)^2} \tag{S3}$$

where $\delta\gamma = \gamma_c - \gamma_0$ is the difference in the uncoupled decay rates. In the main text, we use the approximation of $\Omega_R/2 \approx g$. Figure S7 shows g and $\Omega_R/2$ for a system with various diameters. The smallest diameter, $d = 30$ nm has the highest $\delta\gamma$ and therefore exhibits the largest mismatch between $\Omega_R/2$ and g particularly at low coupling strengths. $\Omega_R/2 \approx g$ is a better approximation for all other diameters. It is worth noting that the onset of the exceptional point occurs when $4g = |\gamma_c - \gamma_0|$ (dotted lines). In the case of the most diluted array of $d = 30$ nm, $\Omega_R/2$ falls below the threshold, but this would result in no splitting, which is incorrect. Therefore, the approximation that $\Omega_R/2 \approx g$ cannot be applied in this scenario. As expected, the calculated value of g is above the threshold.

In Figure 4 in the main text, we could see that the eigenfrequencies did not fully match the minima in reflection for the highest couplings. We have discussed that this is due to the differences between the permittivities used for calculations in FDTD and the TMM. We also mentioned that if the reflectivity and eigenfrequencies calculations were done with the same permittivities, there would be a match between them. This is shown in

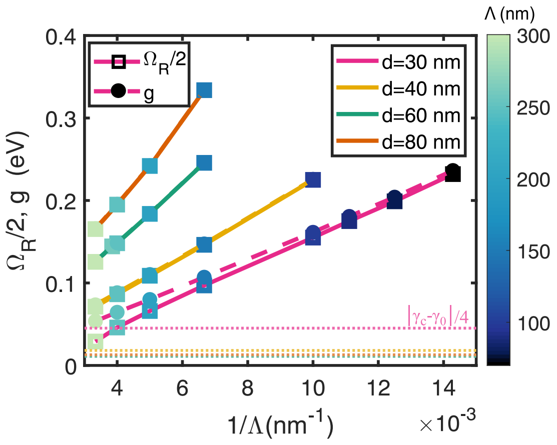

FIG. S7. Comparison of the Rabi splitting and the calculated coupling strength for various array densities $\rho \propto 1/\Lambda$. Data shown for $d = 30, 40, 60$ and 80 nm, $t = 40$ nm, $L = 180$ nm. The dotted lines marks the exceptional point $4g = |\gamma_c - \gamma_0|$.

Figure S8, for various coupling regimes. Particularly for the ultrastrong coupling regime in Figure S8c.

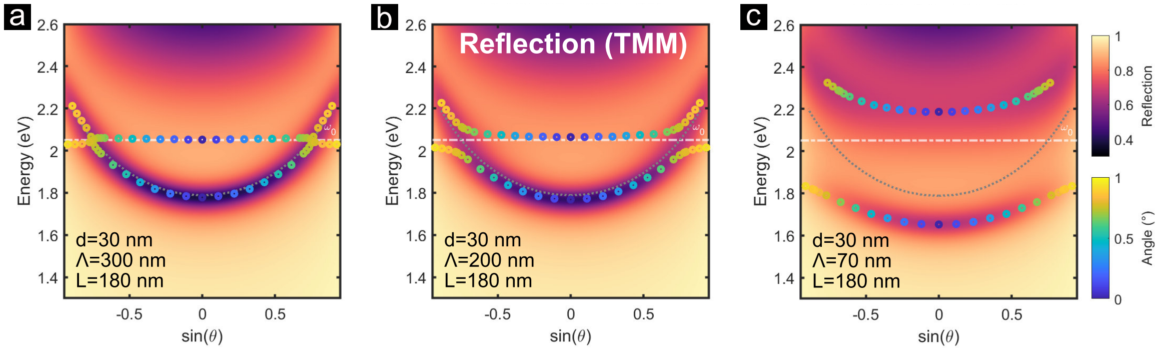

FIG. S8. Examples of poles (QNMs) plotted on top of reflection calculated for various angles by Transfer Matrix Method (TMM). The array is the same as presented in Figure 3 in the main text: $d = 30$ nm, $t = 40$ nm, $L = 180$ nm and the pitches: (a) $\Lambda = 300$ nm, (b) $\Lambda = 200$ nm, and (c) $\Lambda = 70$ nm.

VI. INCREASE OF TOTAL POLARITONIC DECAY RATES AT ZERO DETUNING

Figure 3 in the main text shows that $g \propto \frac{1}{\Lambda}$. Here, Figure S9a, shows the same linearity for all other diameters considered in this article. Despite showing mode splitting, some of the equivalent thin films analyzed in Figure S9a do not reach SC. The dash-dotted lines mark the onset of SC, $(\gamma_c + \gamma_0)/2$, for each thin film associated with a specific nanodisk diameter in the same color. Additionally, shaded in blue are all the arrays reaching USC, whose onset is defined as $\Omega_R/2\omega_0 \approx g/\omega_0 \geq 0.1$ [4].

In the USC regime, the polaritonic eigenfrequencies deviate from a linear behavior with respect to the coupling strength [4, 5]. This deviation starts to be visible in Figure S9b for the largest 80 nm nanodisks, where we plot centered polaritonic frequencies of the system $\omega_\pm - \omega_0$ *vs.* inverse array pitch, $1/\Lambda$. Even though this deviation is minimal for

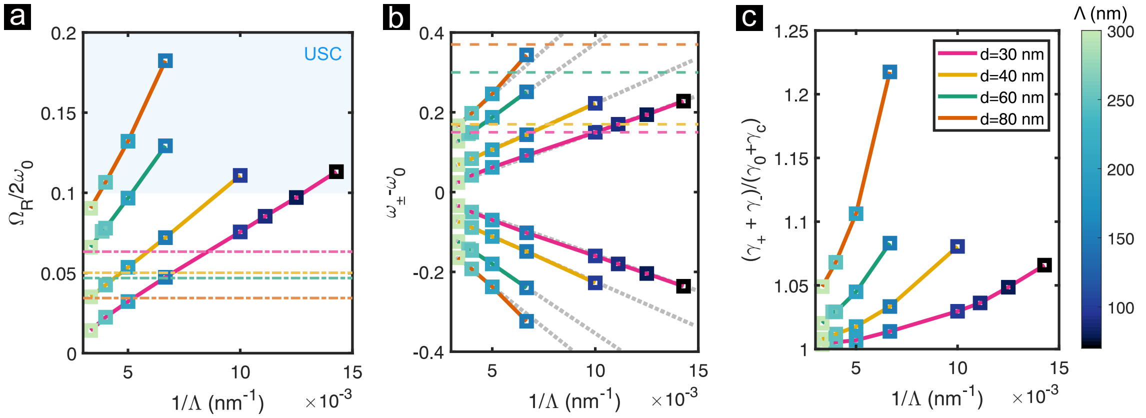

FIG. S9. **Total polaritonic decay rates variation with coupling strength**. (a) Linear dependence of normalized Rabi splitting $\Omega_R/2\omega_0$ on the inverse array pitch, $1/\Lambda$. The USC area is shadowed in blue. The dash-dotted lines mark the onset of SC, $\Omega_R > (\gamma_c + \gamma_0)/2$ for each diameter. (b) Real part of the polaritonic eigenfrequencies dependence on the array pitch. The dashed lines mark the onset of the IBTs at 2.2 eV. Linear guidelines are shown in grey dotted lines. (c) Sum of the imaginary part of the polaritonic eigenmodes normalized by the sum of the uncoupled decay rates shows a non-linear increase with respect to the inverse array pitch for all diameters. For all calculations, $L = 180$ nm and $t = 30$ nm.

real frequencies, the normalized decay rates plotted in Figure S9c show a noticeable non-linear increase. The polaritonic decay rates in the SC regime at zero detuning according to the naive coupled-mode approach are expected to be the average of the cavity and emitter decay rates, $\gamma_\pm = \gamma_{avg} \equiv (\gamma_0 + \gamma_c)/2$ [2, 6]. Yet the data in Figure S9c shows that $(\gamma_+ + \gamma_-) > (\gamma_0 + \gamma_c)$, meaning that the total loss is increasing for all nanodisk – cavity configurations and not only those reaching USC. The main reason for this deviation is the IBTs in gold. As the mode splitting increases with the nanodisk density, the UP approaches the onset of the IBTs (marked in dashed lines in Figure S9b). As a result, the UP experiences additional non-radiative damping. To artificially remove the effect of IBTs, we calculate the eigenfrequency spectra and the corresponding mode splittings in a system incorporating pure Drude gold mirrors, as in Figure S10. This yields $(\gamma_+ + \gamma_-)/(\gamma_0 + \gamma_c) \approx 1$ for all nanodisk diameters except for $d = 80$ nm, as illustrated in Figure S10c, where most arrays are in the USC regime. The increase here is limited to 1.5%, but it may rise further with greater coupling strengths.

VII. POLARITONIC DECAY RATES AT ZERO DETUNING WITH DRUDE MIRRORS

Figure S10 is the analog of Figure S9, but considering gold mirrors that are only described by a Drude model. Therefore, no IBTs are considered.

Figure S10a shows that the linear dependence of the Rabi splitting with the density of the arrays is maintained. In Figure S10b we see the polaritonic frequencies centered with respect to the uncoupled plasmonic response $\omega_\pm - \omega_0$. Only a slight non-linearity is starting to develop for $d = 80$ nm and the densest array.

The main difference with the main text appears for Figure S10c, where the increase of total loss for the polaritons vanishes $\frac{\gamma_+ + \gamma_-}{\gamma_c + \gamma_0} \approx 1$. However, it does increase slightly for the film with the highest oscillator strengths ($d = 80$ nm).

Finally, Figure S10d shows that the upper polariton (UP) decay rate is growing at a similar rate as the lower polariton (LP) one is decreasing. This is what would be expected from the cross-damping model described by Wang *et al.* [7].

VIII. ASYMMETRY OF POLARITONIC RATES AT ZERO DETUNING

Figure 5 considers the average uncoupled decay rates considered for the normalization in a-c contain the uncoupled intrinsic plasmonic decay rate, $\gamma_{avg} = (\gamma_c+\gamma_0)/2$, because the cavity shields the extrinsic (mainly radiative) losses. However, using the FWHM of the plasmonic response in free-space, γ_{pl}, may be tempting because it is readily available from

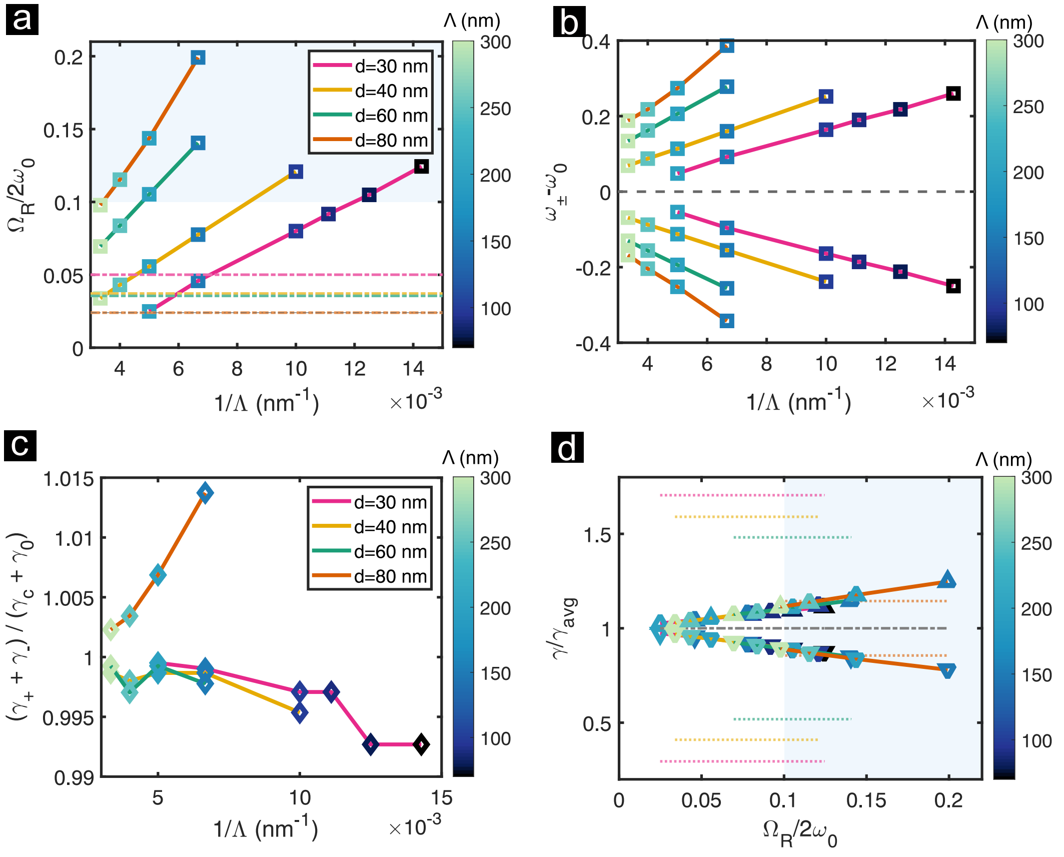

FIG. S10. Eigenfrequencies of all equivalent thin films coupled, at zero detuning, to cavities of $L = 180$ nm and Drude mirrors of $t = 30$ nm. (a) Linear dependence of normalized Rabi splitting $\Omega_R/2\omega_0$ on the array density $1/\Lambda$. The USC area is shadowed in blue. The dash-dotted lines mark the onset of SC, $\Omega_R > (\gamma_c + \gamma_0)/2$ for each diameter. (b) Real part of the polaritonic eigenfrequencies is dependent on the array density. (c) Sum of the polaritonic decay rates normalized by the sum of the uncoupled decay rates. Only the largest diameter shows a minimal increase. (d) Normalized polaritonic decay rates, $\gamma_\pm/\gamma_{avg}$, with $\gamma_{avg} \equiv (\gamma_c + \gamma_0)/2$.

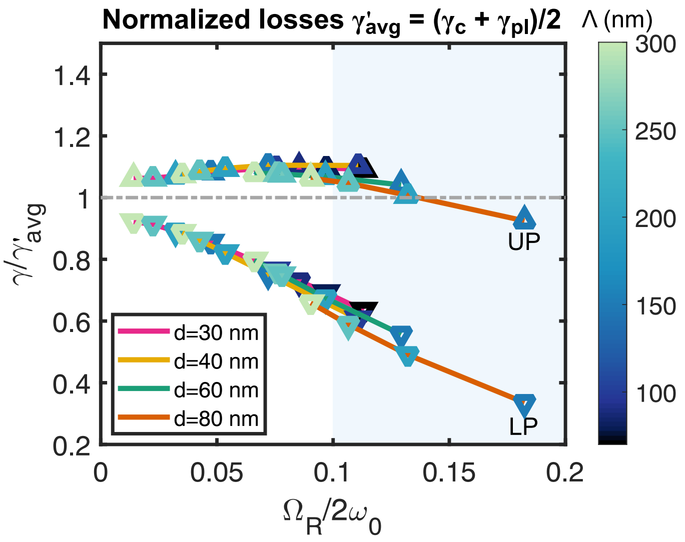

FIG. S11. Same data as in Figure 5c but normalized to the total uncoupled loss, including the meta-atom radiative losses caused by interaction with the environment, $\gamma'_{avg} = (\gamma_c + \gamma_{pl})/2$. γ_{pl} is readily available from the FWHM of the nanodisk array reflectivity outside the cavity.

reflection measurements. In that case, the average uncoupled decay rates increase with the coupling strength as γ_{pl} (marked in dashed lines in Figure 5). Therefore, the normalized polaritonic decay rates change as shown in Figure S11. Interestingly, in such case, both polaritons narrow for the highest coupling strengths $\gamma_{avg} > \gamma_+ > \gamma_-$. Along these lines, it has been suggested that in Deep strong coupling, the decay rates of both polaritons can be suppressed [8]. Here, the narrowing of the LP is more significant because γ_{pl} grows fast with the coupling strength. Nevertheless, it is essential to highlight that γ_{pl} is no longer relevant in the coupled system. The purpose of Figure 5d is solely to demonstrate the effects that can be observed when normalizing to other measurable parameters.

IX. POLARITONIC DECAY RATES AT NON-ZERO DETUNING WITH DRUDE MIRRORS

The frequencies and decay rates of polaritons can be obtained with a non-Hermitian coupled oscillator model [3], as shown in Figure S12, where the polaritons are given by,

$$\tilde{\omega}_{\pm} = \frac{\omega_c + \omega_0}{2} - i\frac{\gamma_c + \gamma_0}{4} \pm \sqrt{g^2 + \left(\frac{\omega_0 - \omega_c}{2} - i\frac{\gamma_0 - \gamma_c}{4}\right)^2}. \tag{S4}$$

As mentioned in the text, within this model, the decay rates are the same as the average of the uncoupled ones at zero detuning, $\gamma_{\pm} = \gamma_{avg} = (\gamma_c + \gamma_0)/2$. At higher detunings, the polaritonic decay rates get closer to the uncoupled ones and are always bounded by

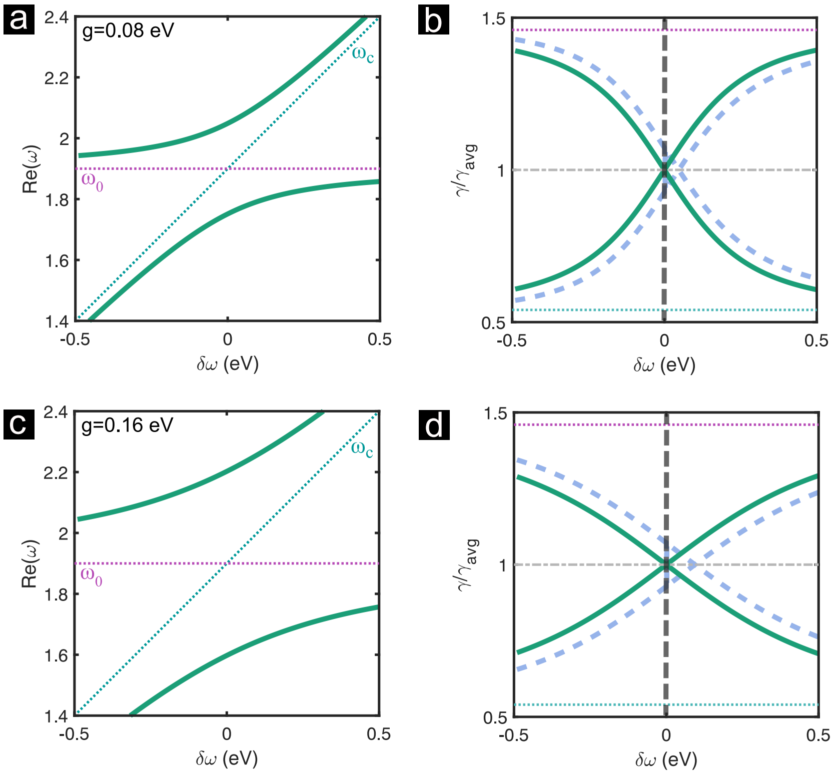

FIG. S12. Eigenfrequencies calculated from a coupled oscillator model at high detunings. (a) Real part of the eigenfrequencies for a $d = 60$ nm, $\omega_0 = 1.9$ eV, $\gamma_0 = 0.2$ eV, $\gamma_c = 0.074$ eV, with a pitch resulting in $g = 0.08$ eV. (b) Normalized decay rate for the same system as in (a). The dashed lines include a cross-damping term as suggested by [7], of $\gamma_{c0} = \sqrt{\gamma_0\gamma_c}/25$. This results in different decay rates at zero detuning. (c) Real part of the eigenfrequencies for a system similar to (a) but with a higher coupling strength $g = 0.16$ eV. Resulting in the same system plotted in Fig. 7a in the main text. (d) Normalized decay rate for the same system as (c), including the dashed line for the decay rates considering the same cross-damping term as in (b).

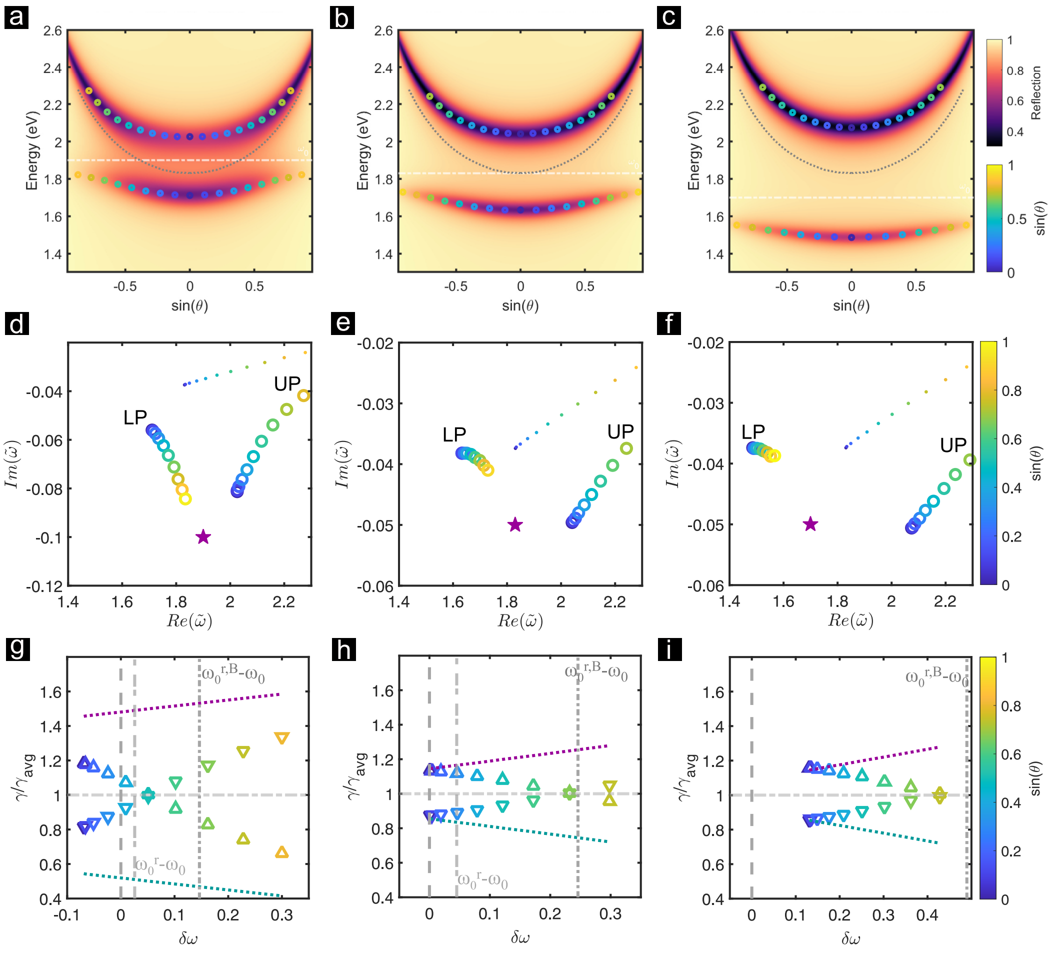

FIG. S13. *Top row:* Angle-dependent reflection spectra calculated via TMM for various equivalent thin films coupled to a $t = 30$ nm and $L = 180$ nm microcavity with Drude mirrors. The real eigenfrequencies are plotted in dots. The thin films had fixed $\Lambda = 260$ nm and diameters: (a) $d = 60$ nm, (b) $d = 80$ nm, and (c) $d = 100$ nm. *Middle row:* (d-f) Eigenfrequencies in the complex frequency plane. The purple stars correspond to the bare plasmon, $\omega_0 - i\gamma_0/2$. The dotted curves mark the empty cavity dispersion. *Botom row:* (g-i) Normalized decay rates variation with detuning. The turquoise and purple dotted lines mark the normalized uncoupled decay rates. Vertical dashed lines mark the detuning corresponding to a cavity sharing the renormalized frequency that considers the coupling strength given by the Rabi splitting, $\omega_0^{r'}$, and the bulk coupling strength, $\omega_0^{r,B}$.

the uncoupled decay rates (in dotted lines in Figure S12b,d).

Figure S12c,d show that when the coupling strength increases, the decay rates at non-zero detuning get closer to γ_{avg}. These figures correspond to the experimental values of Figure 7a in the main text, which is a $d = 60$ nm nanodisk with a pitch of $\Lambda = 260$ nm.

In dashed lines Figure S12b,d we have included the calculation considering a cross-damping term suggested by Wang *et al.* [7]. However, for the calculation in Figure S12d to resemble the data in Figure S13g the cross-damping term was reduced empirically, $\gamma_{c0} = \sqrt{\gamma_0 \gamma_c}/25$.

The main text describes that the decay rates presented in Figure 7 do not follow the behavior described in Figure S12. However, if we consider the mirrors without IBTs as in Figure S13, the decay rates start showing similar behavior to the one described above. In this Figure, the coupling strength increases by increasing the diameter of the nanodisks. Therefore, the bare plasmon and the difference in decay rates vary between columns. Both polaritons share the same decay rate at a higher detuning. Moreover, the higher the coupling strength, the higher the detuning at which the polaritonic decay rates are the same as the average one. This behavior arises directly from Maxwell's equations without considering phenomenological Hamiltonians.

Figure 8 in the main text shows that the detuning at which bulk polaritons have the same decay rate corresponds to a cavity being at zero detuning with the renormalized frequency, $\omega_0^r = \sqrt{\omega_0^2 + 4g_B^2}$. However, in the microcavity, there are other losses that contribute to such detuning not being the needed one to match the decay rates as shown in Figure S13 for Drude mirrors. The lighter vertical dash-dotted line shows the detuning corresponding to the renormalized frequency considering the coupling strength of the system obtained from the Rabi splitting, such that $\omega_0^{r'} = \sqrt{\omega_0^2 + \Omega_R^2}$. None of them match the data due to the losses in the microcavity induced by ohmic losses.

Figure S14 shows the spectra at normal incidence of the nanodisks presented in Figure 7 in the main text. Including a variety of pitches that change the coupling strength. This results in the UP and LP diverging from one another in all the panels, as marked with dashed guidelines.

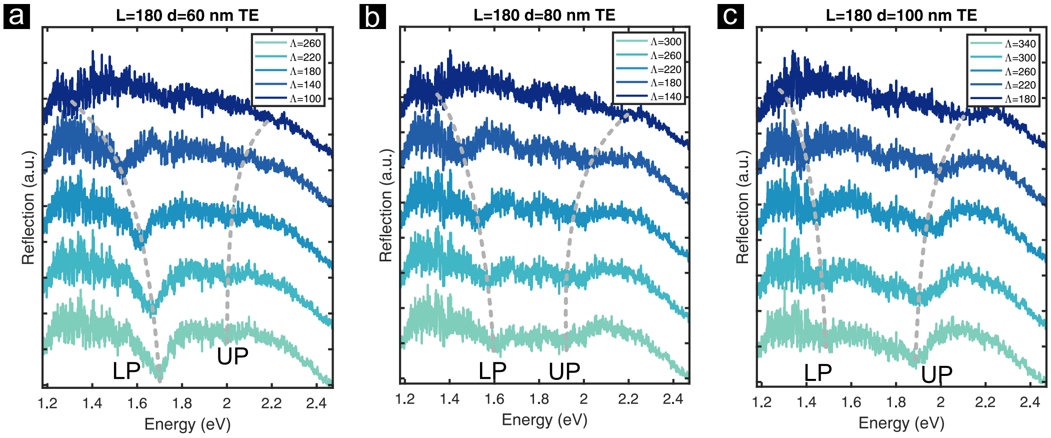

FIG. S14. Experimental normal incidence reflection spectra for plasmonic nanodisk arrays of $\Lambda = 260$ nm coupled to a microcavity of $t = 30$ nm and $L = 180$ nm. The coupling strength increases with the diameter of the nanodisks with (a) $d = 60$ nm, (b) $d = 80$ nm, and (c) $d = 100$ nm.

X. QUALITY FACTORS OF PLASMON – MICROCAVITY POLARITONS

In the main text, Figure 8 showed that bulk polaritons also present an asymmetry in the decay rates at zero detuning, where the LP has a narrower linewidth than the UP. On the contrary, the quality factor $Q = \frac{\omega_\pm}{\gamma_\pm}$ of both polaritons match at zero detuning. This means that both polaritons give the same number of oscillations before being damped. Therefore, the larger the Rabi splitting, the bigger divergence of the frequency of the polaritons will cause a higher asymmetry in the linewidths to compensate.

In bulk polaritons, the decay rates of the uncoupled components are given only by the intrinsic losses of the material, the decay rates are not affected by any other external loss. However, in the case of our plasmon– microcavity hybrid, the cavity introduces radiative losses, and the material of the mirrors introduces non-radiative losses. As mentioned before, the IBTs of gold increase the decay rate of the UP because of its spectral proximity. Thus, a larger Rabi splitting results in the UP being deeper into the IBTs, increasing its decay rate. The LP is further from the IBTs, diminishing its effect on it, which causes an even greater asymmetry in the polaritonic linewidths. Therefore, the quality factors of the polaritons do not balance at zero detuning as shown in Figure S15a-c.

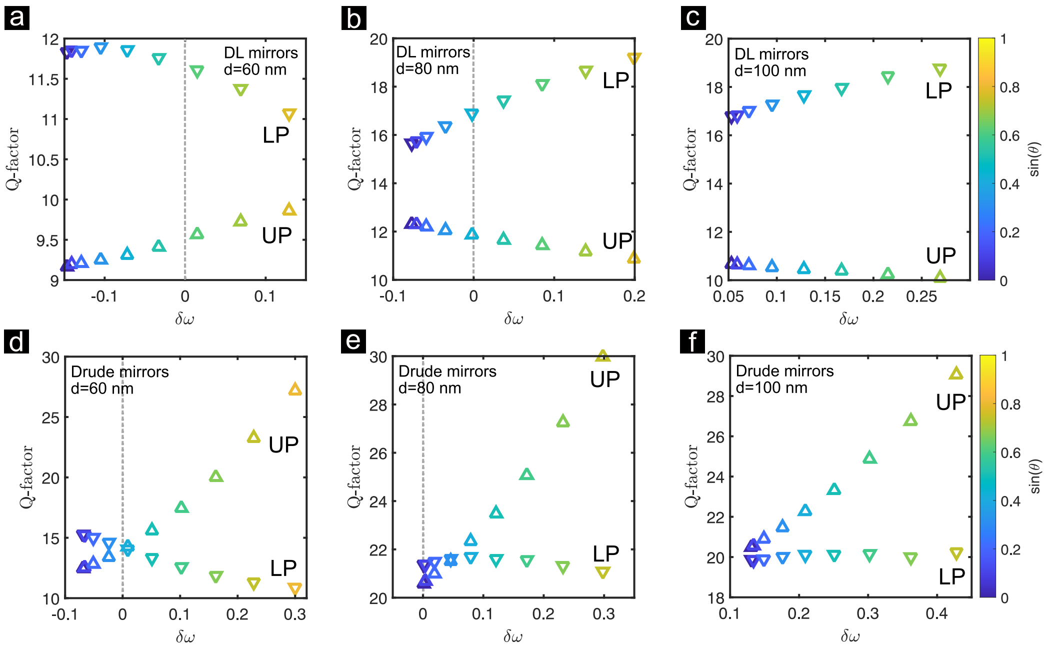

FIG. S15. Quality factor of polaritons with Drude-Lorentz gold mirrors in the *top row* and Drude mirrors in the *bottom row*. All panels have $\lambda = 260$ nm, $t = 30$ nm and $L = 180$ nm with various diameters (a,d) $d = 60$ nm, (b,e) $d = 80$ nm and (c,f) $d = 100$ nm.

As discussed previously, a way to remove the effect of the IBTs is to use a Drude permittivity for gold. Figure S15d-e shows that the quality factors of the polaritons almost match at zero detuning in all cases. However, one must note that using a Drude permittivity still has losses related to the scattering of electrons in the material. Therefore, a small loss is introduced to the system, causing the quality factors to balance almost at zero detuning.

---

[1] Berkhout, A. & Koenderink, A. F. A simple transfer-matrix model for metasurface multilayer systems. *Nanophotonics* **9**, 3985–4007 (2020). URL https://doi.org/10.1515/nanoph-2020-0212.

[2] Törmä, P. & Barnes, W. L. Strong coupling between surface plasmon polaritons and emitters: A review. *Rep. Prog. Phys.* **78**, 013901 (2014).

[3] Baranov, D. G., Wersäll, M., Cuadra, J., Antosiewicz, T. J. & Shegai, T. Novel nanostructures and materials for strong light–matter interactions. *ACS Photonics* **5**, 24–42 (2018).

[4] Frisk Kockum, A., Miranowicz, A., De Liberato, S., Savasta, S. & Nori, F. Ultrastrong coupling between light and matter. *Nature Rev. Phys.* **1**, 19–40 (2019).

[5] Ciuti, C., Bastard, G. & Carusotto, I. Quantum vacuum properties of the intersubband cavity polariton field. *Phys. Rev. B* **72**, 115303 (2005).

[6] Raizen, M. G., Thompson, R. J., Brecha, R. J., Kimble, H. J. & Carmichael, H. J. Normal-mode splitting and linewidth averaging for two-state atoms in an optical cavity. *Phys. Rev. Lett.* **63**, 240 (1989).

[7] Wang, W. *et al.* Interplay between strong coupling and radiative damping of excitons and surface plasmon polaritons in hybrid nanostructures. *ACS Nano* **8**, 1056–1064 (2014). URL https://doi.org/10.1021/nn405981k.

[8] Sergeev, T. T., Zyablovsky, A. A., Andrianov, E. S. & Lozovik, Y. E. Self-consistent description of relaxation processes in systems with ultra-and deep-strong coupling. *arXiv:2303.18159* (2023).
